# A practical guide to pulsed laser deposition

**DOI:** 10.1039/d2cs00938b

**Published:** 2023-03-14

**Authors:** Nick A. Shepelin, Zahra P. Tehrani, Natacha Ohannessian, Christof W. Schneider, Daniele Pergolesi, Thomas Lippert

**Affiliations:** a Laboratory for Multiscale Materials Experiments, Paul Scherrer Institut CH-5232 Villigen Switzerland nikita.shepelin@psi.ch thomas.lippert@psi.ch; b Department of Chemistry and Applied Biosciences, ETH Zürich CH-8093 Zürich Switzerland lippertt@ethz.ch

## Abstract

Nanoscale thin films are widely implemented across a plethora of technological and scientific areas, and form the basis for many advancements that have driven human progress, owing to the high degree of functional tunability based on the chemical composition. Pulsed laser deposition is one of the multiple physical vapour deposition routes to fabricate thin films, employing laser energy to eject material from a target in the form of a plasma. A substrate, commonly a single-crystal oxide, is placed in the path of the plume and acts as a template for the arriving species from the target to coalesce and self-assemble into a thin film. This technique is tremendously useful to produce crystalline films, due to the wide range of atmospheric conditions and the extent of possible chemical complexity of the target. However, this flexibility results in a high degree of complexity, oftentimes requiring rigorous optimisation of the growth parameters to achieve high quality crystalline films with desired composition. In this tutorial review, we aim to reduce the complexity and the barrier to entry for the controlled growth of complex oxides by pulsed laser deposition. We present an overview of the fundamental and practical aspects of pulsed laser deposition, discuss the consequences of tailoring the growth parameters on the thin film properties, and describe *in situ* monitoring techniques that are useful in gaining a deeper understanding of the properties of the resultant films. Particular emphasis is placed on the general relationships between the growth parameters and the consequent structural, chemical and functional properties of the thin films. In the final section, we discuss the open questions within the field and possible directions to further expand the utility of pulsed laser deposition.

## Abstract 2

This tutorial review provides insights on pulsed laser deposition and methods to tune film growth for highly tailored functionalities.

## Graphical abstract

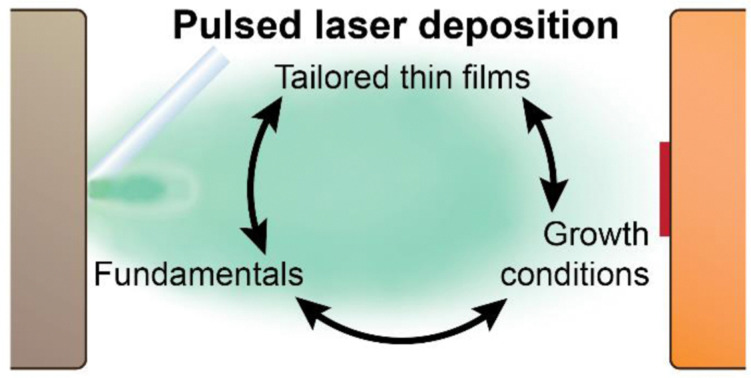

**Key learning points**

(1) Performing the first pulsed laser deposition experiment.

(2) Understanding the dynamics of the laser ablation and growth processes.

(3) Tailoring thin film growth parameters based on experimental observations.

(4) Recognising the utility and advantages of *in situ* monitoring techniques.

(5) Correlating the properties of the growing film to those of the ablated species.

## 1. Introduction

Thin films are tremendously useful for practical, functional and fundamental applications already covering large aspects of our daily lives. The literature reports countless examples of physicochemical bulk properties that change, sometimes drastically, when prepared as thin films. In some cases, thin films possess properties not present in the bulk. The resultant functionalities can be magnetic, electrical, anti-corrosive, reflective and anti-reflective, hydrophilic and hydrophobic, oleophilic and oleophobic, selectively light absorbing, and antibacterial, among many others. These thin films can produce functional behaviours in a small size, which has resulted in tremendous advances in the field of electronic materials, namely in the use of thin film transistors, semiconductors, solid state batteries, electrocatalysts and photoelectrocatalysts, sensors, and precision actuators. Additionally, thin films are often ideal model systems to characterise material properties, which are difficult or impossible to probe otherwise. A typical example is the characterisation of interfacial properties. Due to the possibility of tailoring a surface to obtain a sharp interface between the substrate and film, or between two films, unique nano-architectures can be fabricated in the form of multilayered heterostructures.

A multitude of techniques can be employed in order to deposit thin films. These are commonly divided into two subsets, chemical deposition, and physical deposition, based on the route taken to produce the film. Both routes are generally capable of achieving atomic-scale precision of the resultant films and are therefore referred to as bottom-up mechanisms, as they follow a process of material growth on a substrate material. The chemical deposition route commonly utilises precursor materials, which are either in a liquid or in a gaseous state. The particulars of this route have been detailed elsewhere^1–3^ and readers are directed to these for an overview of chemical-based deposition techniques. In comparison, the physical deposition route utilises solid precursors. A thermodynamic (*i.e.*, energetic) approach is employed for the atoms in the precursor to eject from the surface and travel toward the substrate. This physical deposition route forms the basis of this tutorial.

Within the subset of physical deposition, multiple techniques exist, disparate in the methods they employ for the ejection of atoms from the precursor material. One particularly useful physical deposition method is pulsed laser deposition (PLD), where the energy of a pulsed laser is directed towards a target, in order to vaporise and dissociate the target material.^4^ The ejected species form a dense cloud of material, which further interacts with the initial laser pulse and the ambient gas to form a highly excited plasma through scattering, photoexcitation or multiphoton absorption processes. This process produces charged layers (*i.e.*, electrons, as well as light and heavy plasma species) that form a field, accelerating the plasma species to energies between a few electron volts and thousands of electron volts. A mixture of ions and neutrals with a broad range of kinetic energies will eventually reach the substrate, where they form a film. A suitable gaseous environment, at different partial pressures, can be set in the deposition chamber to allow physical and chemical interactions. This technique is incredibly powerful and precise, enabling fine tuning of deposition parameters, which leads to a high degree of control over material properties. It is also the only technique that allows for deposition at a wide range of pressures, from ultra-high vacuum to the mbar range. Among all available physical deposition methods, PLD offers the broadest range of available material compositions and is the most effective technique for materials with complex chemistries. However, this technique can seem extremely complicated, due to the vast range of parameters that must be controlled. This tutorial has been written on account of this complexity in PLD, breaking down the critical requirements with the aim of enabling researchers new to the field to perform their first experiment with a practical understanding of the system.

This tutorial review is divided into three parts representing the varying layers of complexity within the experimental method. Section 2 discusses, in greater detail the operational principles of PLD, including an overview of the instrumentation used. Subsequent parts of this section provide an overview of the fundamental processes occurring throughout the laser ablation process, the specifics of the plasma that forms as a result of the ablation, and finally how the nucleation and growth of the resultant film proceeds. Section 3 breaks down the key parameters that can be tailored to obtain the greatest extent of control in PLD. This section describes the influences of the parameters relating to the laser, gaseous environment, substrate and target, along with their subsequent effects on film quality and functionality. The final part of this tutorial outlines the techniques used during the growth of the films to monitor their quality *in situ*. This section covers the use of reflection high-energy electron diffraction (RHEED) as a way to monitor the growth mechanism; multi-beam optical stress sensor for the measurement of in-plane stresses; and mass spectrometry and plasma imaging to obtain insights on the aspects of the PLD process, ranging from plasma expansion to film growth and compositional analysis.

## 2. Fundamentals of pulsed laser deposition

The processes constituting PLD are complex in nature, requiring a multifaceted understanding of the constituent mechanisms. As part of this, it is imperative to familiarise oneself with the components of the PLD instrumentation, as well as the three critical stages of the PLD process, namely laser ablation, plasma formation, and film nucleation and growth mechanisms. This section briefly outlines these aspects, which will form the foundation for the further concepts discussed in this review.

### 2.1. Overview of instrumentation

**Fig. 1:**
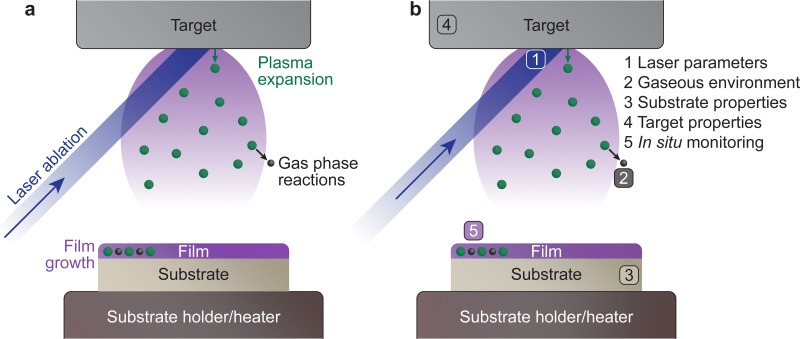
(a) An overview of the key processes involved in pulsed laser deposition. In this technique, a pulsed laser ablates a target surface, ejecting the species from the target in a highly energetic state, which forms a plasma. During the travel of these species from the target to the substrate, they can undergo gas phase reactions with the surrounding atmosphere, and consequently reassemble on the surface of the substrate in the form of a thin film. (b) The main parameters tailored during the pulsed laser deposition process, which will be discussed as part of this tutorial review.

The PLD system is composed of a vacuum chamber with at least one window possessing transparency to the incoming laser wavelength. Within the vacuum chamber, two main components must be present: the target and the substrate holder (Fig. 1(a)). These two components are separated by a predefined gap, such that the surface of the target faces the substrate holder. A laser is used to ablate the material of the target, which creates a plasma that expands towards the substrate in what is commonly known as the “plasma plume”. This plasma corresponds to the energetic species, which deposit on the substrate and form the film. The experimental variables of the PLD process are summarised schematically in Fig. 1(b).

#### 2.1.1. Laser

The laser forms a vital part of the PLD process and thus its influences must be considered for this experimental technique. The word “LASER” is an acronym, which expands to light amplification by stimulated emission of radiation. In this section, we will succinctly describe the working principle of a laser.

**Fig. 2:**
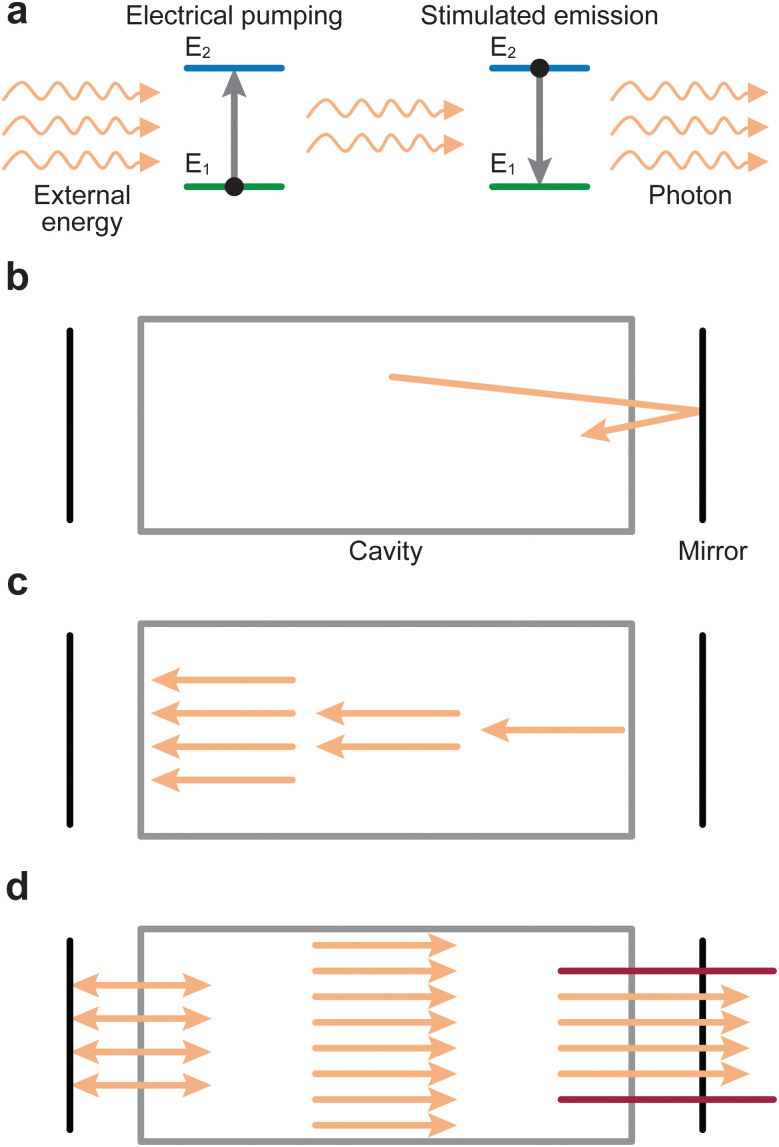
Simplified schematic representation of the processes occurring inside a laser. (a) The medium absorbs energy from an external source, through a process called electrical pumping, emitting photons as a result. (b) Electrons cycle between the two energy levels (E_1_ and E_2_) due to the applied energy, releasing the energy as monochromatic light. (c) The intensity of the photon beam is amplified by a series of mirrors. (d) The mirrors direct the monochromatic light as a laser beam at the exit.

An electron can move into an excited state (E_1_ to E_2_ in Fig. 2(a)) provided it receives a particular amount of energy. Subsequently, during the relaxation process, this excited electron releases the surplus energy in the form of photons (*i.e.*, light). Generally, the electrons relax to a lower energy level quickly following excitation, which results in random emission of photons (incoherent emission). It is also possible for electrons to populate an excited state longer and relax together into a lower energetic state (coherent emission). The first process is called spontaneous emission and is a random process, whereas the second process is called stimulated emission and produces photons with identical properties (phase, amplitude and frequency). More importantly, this is a multiplicative process. To make use of this property, a laser is therefore composed of an optical medium as part of a resonant optical cavity, where the constituent species are promoted to an upper excited level through either an applied electrical field (commonly referred to as electrical pumping) or an optical excitation by either a flash lamp or another laser. The relaxation of the system results in the emission of a significant number of photons, which occurs as the electrical energy is removed (Fig. 2(b)). These photons undergo amplification through a set of mirrors in resonance with the emitted photon wavelength, thereby directing the photons back and forth within the cavity (Fig. 2(c)). Finally, the photons are released from the cavity *via* a semi-transparent window and focused into an aperture (Fig. 2(d)).

The most common lasers used for PLD are gas (excimers, or excited dimers; electrically pumped) or solid-state lasers (optically pumped), for example the higher harmonics of an yttrium aluminium garnet (YAG) laser due to their high output power. Table 1 summarises the properties of commonly utilised lasers. The pulse length affects the laser ablation process and determines how much thermal effects dominate. Most PLD systems employ nanosecond lasers possessing a laser pulse length between 10 ns and 25 ns, which has proven over time to be the optimal window with respect to plasma properties and oxide thin film growth. The pulse shape and repetition rate can also affect film growth.^5–7^ Both of these effects are discussed further in Section 3.1.

**Table 1:** Summary of the laser types commonly employed in PLD

Laser type	Medium	Wavelength (nm)	*E* (eV)
Solid state	YAG ω	1064	1.16
Solid state	YAG 2ω	532	2.33
Solid state	YAG 3ω	355	3.49
Solid state	YAG 4ω	266	4.66
Excimer	XeCl	308	4.02
Excimer	KrF	248	4.99
Excimer	ArF	193	6.41

#### 2.1.2. Vacuum chamber

**Fig. 3:**
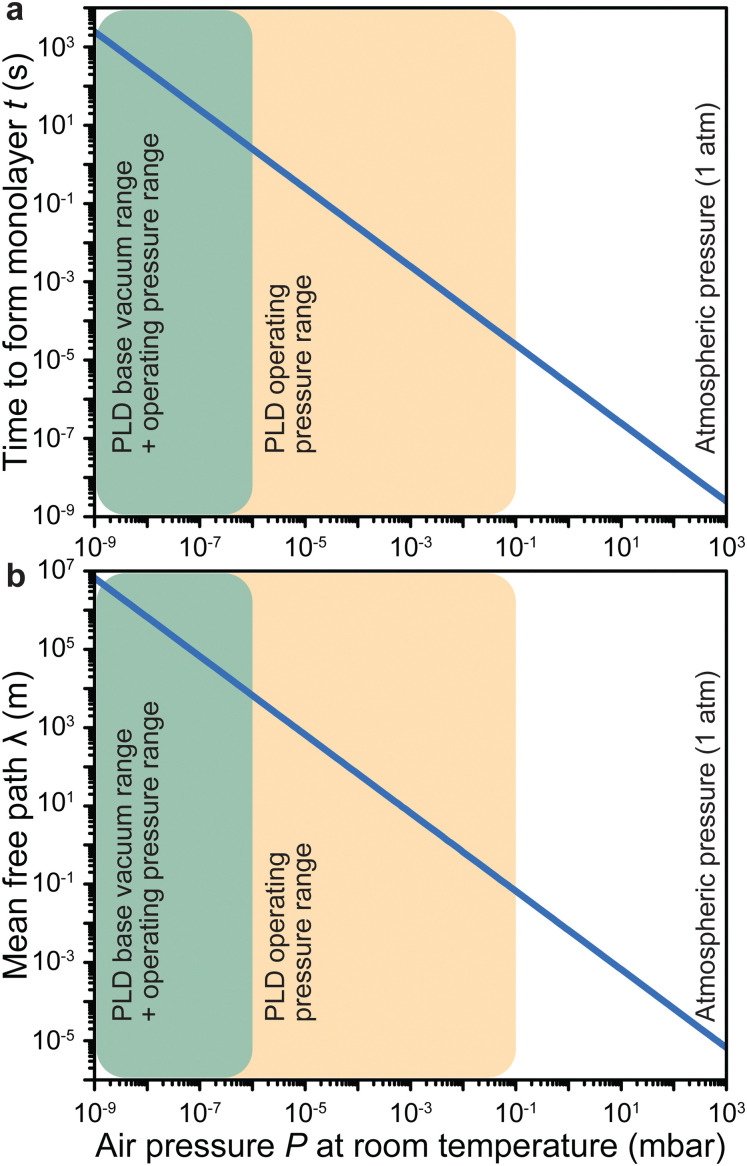
The impact of air pressure at room temperature on (a) the time to form a monolayer of adsorbate species that are present in the vacuum chamber and (b) the mean free path. The yellow overlay corresponds to the pressure range over which deposition of films has been reported. The green overlay corresponds to typical base pressures of pulsed laser deposition. Film growth can also proceed in the latter range.

The vacuum in the chamber is achieved by a series of turbo molecular and rough pumps. The base pressure in vacuum chambers used for PLD is generally between 10^−6^ mbar and 10^−9^ mbar. Without considering other sources of contamination in the chamber, the vacuum level determines how fast a single surface layer of contaminants and impurities can form on the surface of the substrate. This time *t* can be estimated by eqn (1).1
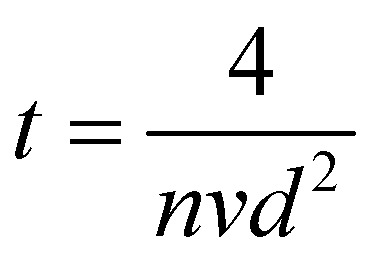
Here, *n* corresponds to the number of molecules per unit volume, *v* the average velocity of the molecules, and *d* the diameter of a molecule. In air, eqn (1) can be simplified to the form shown in eqn (2), with *P* representing the air pressure in units of mbar. This equivalence is demonstrated in Fig. 3(a).2
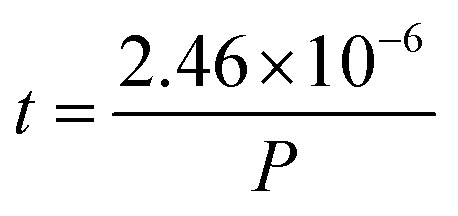


Under low vacuum conditions (*i.e.*, 0.1 mbar), a layer of contaminants forms in a few microseconds. However, reducing the pressure provides a more stable environment whereby fewer contaminants are present in the chamber, thus drastically increasing the timeframe for the formation of a contaminant layer.

How far a molecule travels before it collides with another molecule is defined as the mean free path. The level of vacuum affects the mean free path, *λ*, of the species present in the chamber and thus the entire plasma dynamics. The mean free path is characterised by eqn (3a). In air at room temperature (23 °C), it can be further simplified as shown in eqn (3b) and in Fig. 3(b).3a
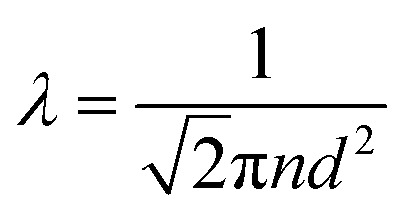
3b
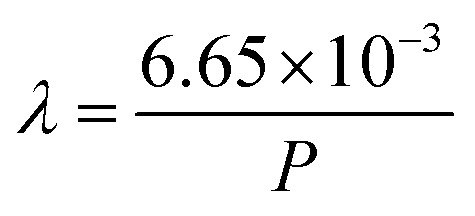


As shown in Fig. 3(b), the environmental pressure drastically influences the mean free path, ranging in scale from micrometres to kilometres by decreasing the pressure from atmospheric values down to 10^−6^ mbar.

#### 2.1.3. Target holder

**Fig. 4:**
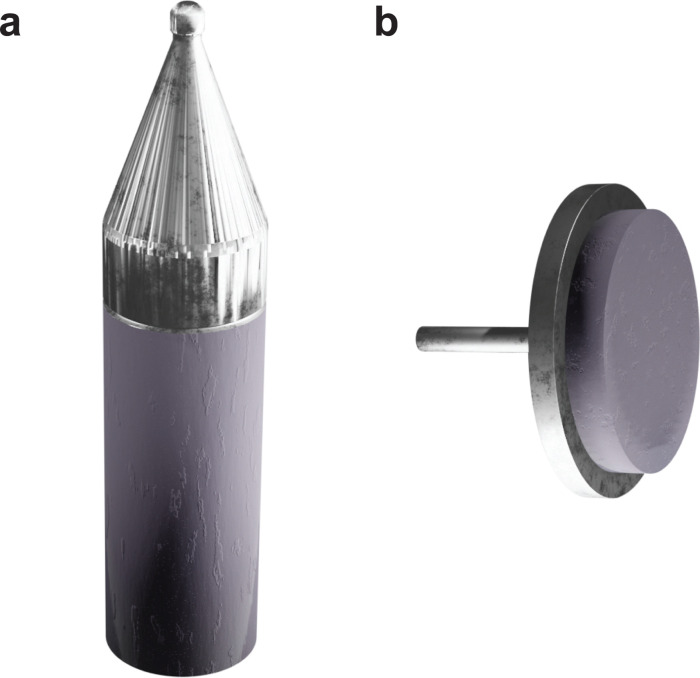
The respective geometries of the two common target holder types, (a) rod-shaped holder and (b) disk-shaped holder.

There are a number of different target shapes, which are used for ablation. All of them offer certain benefits, but also hold drawbacks dependent on the goals of the experiment. The two common types of targets are either rod-shaped (Fig. 4(a)) or thin plates in the form of a disk (Fig. 4(b)). Common to all targets, the location of the laser beam on the target must be moved to avoid material removal-induced cratering of the surface. As the optical path of the laser is usually fixed, the target is moved throughout the deposition.

The rod-shaped target holder (Fig. 4(a)) is typically mounted to a spindle, which enables the rotation of the target and an automated height modulation between two limit switches. These movements are necessary to ensure homogeneous ablation of the entire target surface area. The primary advantage of this geometry is its mechanical simplicity. With this geometry, multilayered heterostructures can be fabricated by stacking rods of different materials on top of each other. Conversely, this type of target holder is sensitive to the positioning of the target. The rotational motion can induce lateral shifts if the target is not centred properly or if the side walls are corrugated, which affects the position of the laser spot and subsequently can alter the direction of the plume with respect to the substrate position. Considering the laser spot typically possesses a rectangular shape at the target surface, alignment of the long axis of the laser spot with the long axis of the target can result in a higher degree of thickness homogeneity, with large area coatings on substrates up to 20 cm × 20 cm demonstrated in literature.^8–10^

The main drawback of the rod-shaped target is that the poor sintering properties of many materials make the fabrication of sufficiently long rods very challenging and sometimes impossible, whereas disk or plate shaped targets (Fig. 4(b)) are generally easier to prepare.

In contrast to the movement of the rod target, the disk target is either rastered or rotated. The rastering path can be customised to any combination of *x*–*y* axis movements. A combined rotation/rastering motion is also possible.

#### 2.1.4. Heater and substrate holder

A high substrate temperature is one of the requirements for the growth of crystalline materials. Hence, the substrate holder is part of a heating system, allowing film growth at temperatures of up to 1000 °C. These are mostly resistive heaters. Recently, laser based heating set-ups have become more common, offering an increase in flexibility for heating ramps and deposition temperatures up to 1500 °C.^11–13^ The geometry of these components impacts the film growth, contributing to many disparities between PLD systems and thus inconsistencies in material growth between differing chambers. For example, the geometry of the heater determines the size of the heated zone, and therefore the heated volume in the chamber, including the temperature at the target surface. Changes in heated volume in front of the substrate can affect the local density of the incoming plasma and consequently modify the growth process. It is common to observe a colour change of an oxide target material during ablation when using a small-area heater, which occurs due to the removal of oxygen. Conversely, depositions utilising a large area heater in an oxygen atmosphere allow sufficient heat transfer to re-oxidise the target during ablation.

The size and shape of the heater, as well as how the thermal contact between heater and substrate is ensured, are among the main reasons for the relatively wide range of deposition parameters reported in literature for the same material.

### 2.2. Laser ablation process

Armed with an understanding of the instrumentation that forms a PLD instrument, we will now take a closer look at the chemical and physical phenomena that occur during the process of PLD. The fundamental processes that form part of PLD are the key to engineering thin films. These processes are divided into three groups: (1) laser ablation; (2) plasma formation and the expansion of target material; and (3) subsequent nucleation and growth of the target material on the substrate. These concepts are complex and are deserving of their own discussion, therefore readers are directed to the excellent reviews by Schou,^14^ and Wilmott and Huber^15^ for further detail. In the following section, we will provide a short discussion on the phenomena occurring throughout each of these three stages.

#### 2.2.1. Laser absorption

Generally, in order to be physically removed, the target material must be able to absorb the applied wavelength. However, this argument does not fully hold in the case of ultra-short laser pulses for material removal. In this case, laser ablation proceeds *via* thermal and non-thermal processes, and is often a combination of both. These processes take place on different time scales; however, the combination of both contributes to the formation of a plasma plume.

**Fig. 5:**
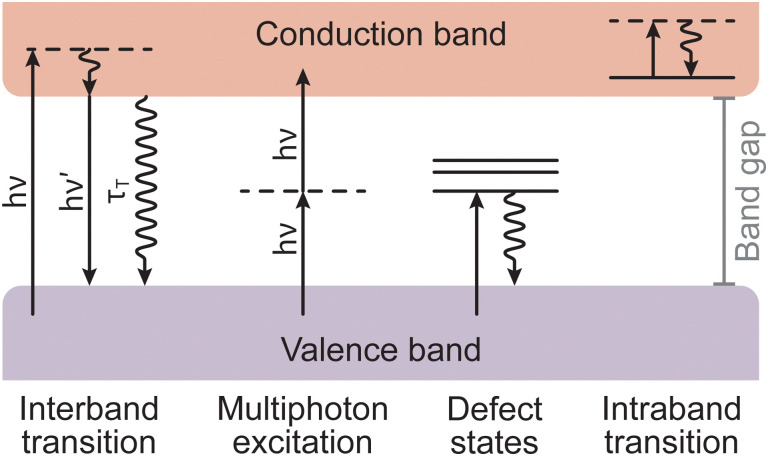
Schematic of different types of electronic excitations in a solid. Straight arrows represent the absorption or emission of photons of energy *hν*, while oscillating arrows represent non-radiative processes with *τ*_T_ as the respective relaxation time.

Non-thermal interactions dominate the first stage of the laser ablation, whereby incident photons provide enough energy to induce band transitions as the target material absorbs the light (Fig. 5). The main non-thermal absorption mechanism takes place *via* the excitation of an electron by an incoming photon. The electron undergoes an intraband transition from the ground state to an excited state and the excited electron relaxes through electron–phonon coupling. It is also possible to have an excitation of one molecule by two incoming photons. When this process occurs within at the picosecond timescale or shorter, the energy of the two photons is combined to promote the molecule into an excited state. This is called multiphoton excitation and can be either coherent or sequential. The properties of the material can affect this absorption process as, for instance, the presence of defects can modulate the light absorption properties (*i.e.*, the band gap).

The extent to which the photons propagate through the material can be calculated by the Beer–Lambert law (eqn (4)), where *A* is the absorbance, *I* the light intensity, *I*_0_ the incident light intensity, *ε* the molar extinction coefficient, *c* the molar concentration and *t* the thickness. Eqn (4) can thus be used to calculate the penetration depth of the absorbed laser light and estimate the extent of the non-thermal processes.4
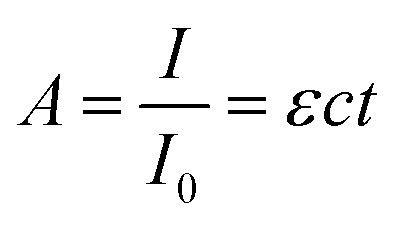


The second type of interaction is thermal excitation. One of the main attributes of laser-induced thermal excitation is the rapid heating in a localised area to temperatures exceeding several thousand Kelvin. The vibrational relaxation of excited electrons (electron–phonon coupling) transfers heat to the system on a picosecond timescale.^16^ The typical PLD pulse duration of approximately 20 ns is therefore long enough for thermal processes to dominate. As part of these processes, the surface heat will propagate into the bulk of the target, which can cause unintended side effects (*e.g.*, mechanical stress, micro-crack formation, solidification and remelting, among others). The thermal diffusion length *L*_T_ can be calculated by eqn (5), taking into account the thermal diffusivity *D* and the laser pulse length *τ*_L_.

5 *L*_T_ = (2*Dτ*_L_)^1/2^

**Fig. 6:**
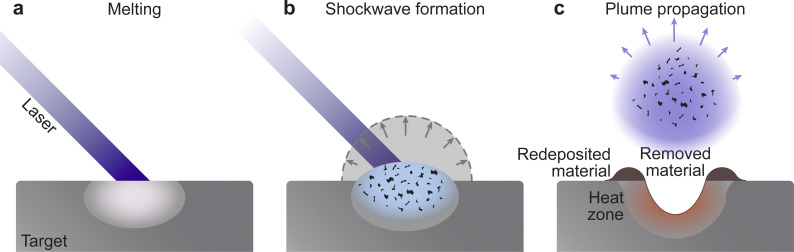
Schematic demonstrating the processes occurring during laser ablation, which lead to the expulsion of material from the target: (a) melting and instantaneous vaporisation, (b) shockwave formation and (c) plume expansion after the termination of the laser pulse.

Consequently, the material is ejected from the target following a combination of electronic and thermal ejection mechanisms (Fig. 6). The exposure of a molecule to excess electrons causes instability, which results in its desorption, fragmentation, or ionisation from the target surface. Hence, the thermal ejection following laser impact is the result of heat-induced bond cleavage through evaporation or bond dissociation. This process is further aided by intermolecular collisions, leading to excitations and ionisation in the very dense vapour, while still interacting with the incoming laser pulse.

### 2.3. Plasma formation and expansion

#### 2.3.1. Initial vapour formation

The absorption of the laser light is not limited to the solid target material, occurring also in the initial vapour of the removed material. In the vapour region, the energy is primarily absorbed by either photoionisation or inverse Bremsstrahlung. Photoionisation corresponds to electron ejection from an atom following photon absorption, whereas inverse Bremsstrahlung occurs when an electron gains kinetic energy by a collisional energy transfer with other particles. These mechanisms promote the generation of free electrons, which, in turn, increase the degree of ionisation of the plasma.

The frequency of collisions increases with an increasing density of the vapour, forming the so-called Knudsen layer. This layer is a key driver for the consequent expansion mechanism of the plasma plume. It is a near-surface region with the thickness corresponding to a few multiples of the mean free path, where the ejected species have a strongly forward-peaked velocity distribution away from the sample surface. Depending on the density of the layer, the plasma plume will expand first in a collisional manner, contributing to the high degree of ionisation and charge separation. This is followed by an adiabatic expansion and a free flight of plume species for an ablation with a background pressure equivalent to a large mean free path. For a background pressure considerably shorter than the target-substrate distance, a shockwave is formed thereby moderating the kinetic energy of the plasma species (Fig. 6(b)).

#### 2.3.2. Plasma formation and early-stage expansion

The initial plasma expansion is characterised by a charge separation, with chemical species being accelerated based on their mass. Electrons and light elements lead the plasma plume expansion, while heavy species remain at the back. This separation, based on the charge to mass ratio, creates an electrostatic gradient that is equivalent to an electrostatic double layer. Electrons, the lightest and fastest species, are split into two types: the energetic electrons (called the hot electrons) are situated at the front of the plume, while the cold electrons trail at the back, well behind the fast ions.^17^ The resulting electric field gradient accelerates the ions inside the double layer, thus the time spent in this region determines the maximum kinetic energy of the species. The presence of the double layer affects the expansion dynamics of both heavy and light species, and subsequently the number of species reaching the substrate.^18^

### 2.4. Film nucleation and growth

The growth of a thin film proceeds through a series of steps. Arriving species either impinge on the surface, diffuse and begin the nucleation process, bounce, or desorb. These processes are temperature and deposition rate dependent. This section briefly describes the diffusion, nucleation and coalescence phenomena.

#### 2.4.1. Surface diffusion and nucleation

Upon reaching the substrate, the species forming the plasma plume can physisorb and further chemisorb. The mode of adsorption depends on the effective coverage of the surface. At low coverage, the species follow tracer-like diffusion, whereby atoms hop between energetically favourable sites of the substrate surface. An increased coverage results in the dominance of Coulomb interaction between neighbouring atoms, leading to a chemical diffusion mechanism. On uniform surfaces without chemical traps, the adsorbing atoms diffuse intrinsically, while the presence of defects pushes the adatoms to adopt a mass transport diffusion behaviour. Mechanistically, the motion of adatoms occurs *via* hopping between favourable sites, tunnelling through energetically favourable paths, atomic exchange, or diffusion through vacancies.

The formation of clusters is influenced by the surface tension of the surface. In the case of thin films, heterogeneous nucleation allows the atoms to aggregate up to a critical radius. The nuclei are stable and continue to grow if they exceed the critical size, otherwise they will dissociate.

#### 2.4.2. Coalescence

Coalescence of the growing film can be done *via* Ostwald ripening, sintering or cluster migration. The common driving force of these mechanisms is the reduction of the Gibbs free energy, which occurs through an overall shrinking of the surface area.

The growth mode is based on the binding interactions exerted within the film and the substrate. It is defined by how the species diffuse at the surface. Among all growth modes, there are three standing out:

• Volmer–Weber or island growth: the high surface tension resulting from the high atom–atom interactions compared to the atom-surface interactions causes the film to grow in island-like formations. It has to be noted that the shape of the islands can differ.

• Frank–Van der Merwe or layer-by-layer growth: the interactions between the atoms and the surface are stronger than between the atoms themselves.

• Stranski–Krastanov or layer and island growth: the film starts growing in a layer-by-layer mode. Because of external forces (*e.g.*, lattice mismatch), the growth is disturbed and island-like nucleation is favoured again.

## 3. Parametric influences in pulsed laser deposition

PLD is a highly versatile technique, allowing the deposition of a wide range of materials. The versatility of PLD is evidenced through the deposition of films and structures with highly tailored properties, such as the structure (polycrystalline, epitaxial or coherent with a broad range of crystallographic space group combinations), composition (stoichiometric, off-stoichiometry, or doped), and even density (ranging between the maximum values for the crystalline material and ultra-low densities resulting in foam-like structures). Nonetheless, the behaviour of the vaporised species leaving the target following ablation, as mentioned in Section 2, is complex and depends on a range of variables. Often, the deposition parameters for thin films with varying compositions can be found in literature from prior experiments. These literature values tend to form the basis for subsequent experiments, although it is important to understand that optimal values in one chamber do not necessarily result in the same film quality when used in a differing chamber. Thus, an optimisation procedure is performed in order to tailor the parameters to the chamber used. In this section, we discuss the key variables in the formation of thin films. The influences of parameters relating to the laser, the gaseous environment, the substrate and the target will be described in detail.

### 3.1. Laser

In PLD, the characteristics of the laser beam are of the utmost importance as they rule the ablation mechanism. Understanding how the laser beam affects the ablation process is critical to achieve the desired control on the deposition process.

#### 3.1.1. Wavelength selection

The light absorption properties (*i.e.*, the band gap) of a material determine the laser wavelength to be used for the PLD process. Common laser wavelengths PLD users select are ArF (193 nm, or 6.42 eV), KrF (248 nm, or 4.99 eV), XeCl (308 nm, or 4.03 eV), and Nd:YAG (1064 nm, or 1.16 eV). Ablating metals with an ultraviolet (UV) laser is difficult, as the electrons from the conduction band shield the material from the UV radiation and reflect most of the laser light. Metal ablation has been successfully accomplished, albeit at an intrinsically lower ablation rate.^19–21^ The UV ablation of metals is largely a photothermal process and very inefficient. Oxides absorb photon energy in the UV range, with the 248 nm (KrF excimer) being the most commonly used wavelength. This wavelength combines a number of advantages, namely a reasonably long lifetime of the gas mixture, little absorption in air (unlike an ArF laser), and, with an energy of 4.99 eV per photon, enabling the dissociation of most chemical bonds in a solid through a single-photon absorption process. There are only a handful of oxides where a wavelength shorter than 248 nm is preferable, for example MgO, Al_2_O_3_ or SiO_2_ (quartz); however, *λ* = 248 nm often works on sintered targets of these materials.

A typical energy that binds the atoms in a non-metal is about 2–5 eV.^22^ By selecting the laser wavelength (photon energy), one can tune which bond to preferentially cleave. This is also reflected when measuring the species composition of the ejected ablation plume. For nanosecond ablation with *λ* = 248 nm and 193 nm, the plume consists largely of single atomic plasma species with a few binaries or even ternary metal–oxygen species, while an ablation with *λ* = 308 nm results in the prominence of binary metal–oxygen species as part of the overall plume composition.^23^ Consequently, the film formation on a substrate will somewhat depend on the laser wavelength used, since the starting composition of the arriving species on the substrate differs, which will affect the diffusion of species on a substrate surface, together with the initial cluster and subsequent film formation. This has been evidenced through wavelength-dependent studies of the deposition of AlN and ZnO, whereby both studies demonstrated improved film quality at lower wavelengths due to the relatively large band gaps of the targets.^24,25^

#### 3.1.2. Pulse length and pulse energy

**Fig. 7:**
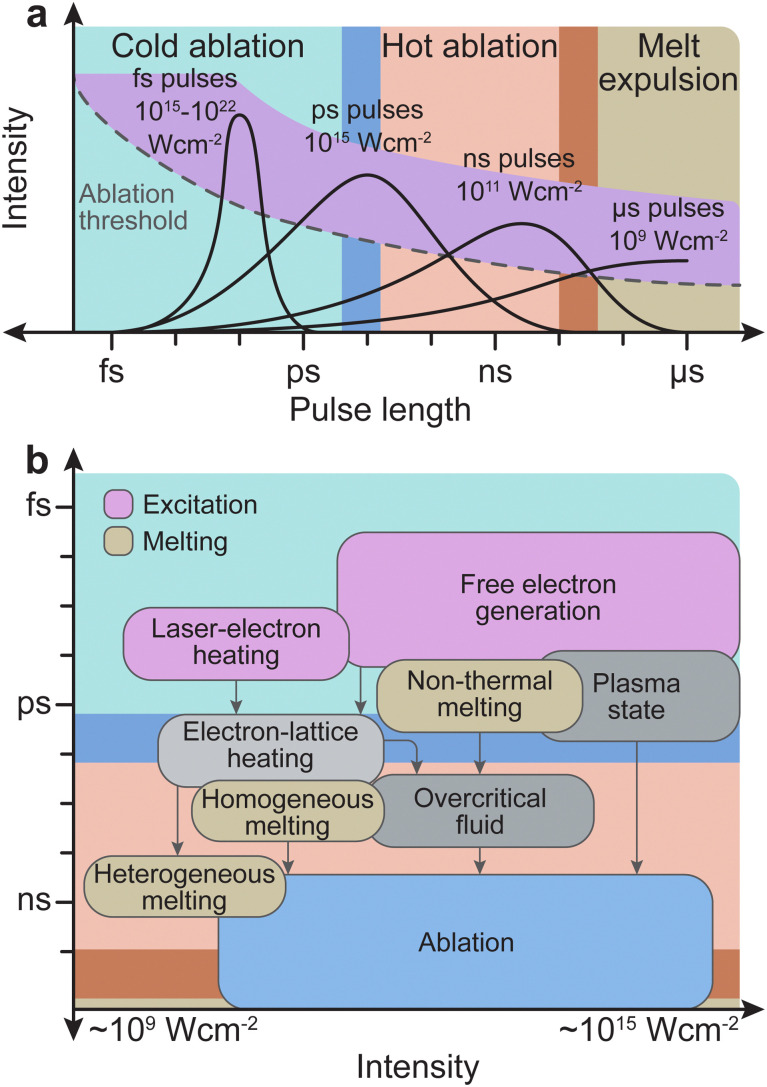
(a) The three physical domains: cold ablation, hot ablation and melt expulsion, with respect to the corresponding time scales for the laser pulse length. The ablation threshold (dotted line) and the maximum available pulse energy (purple overlay) are qualitatively indicated. For long pulse durations, the radiation further interacts with the ablated material in the vapour phase. (b) Typical timescales and intensity ranges of several phenomena and processes occurring both during and after irradiation of a solid with an ultrashort laser pulse of about 100 fs duration. Excitation takes place in the range of femtoseconds (duration of the laser pulse). The timescale of melting may vary for different processes and lies roughly in the picosecond regime. Material removal (*i.e.*, ablation) occurs in the nanosecond regime.

The initial stages of the interaction between laser light and the solid target are crucial. The energy density per pulse plays a strong role in this process, which is governed by the pulse length and the pulse energy (Fig. 7(a)). The typical timespan necessary for material ejection and plume formation is on the order of tens of picoseconds, although this timespan is also dependent on the energy; however, further interactions are necessary between the laser light and the species in the plasma plume. It is also important to register if the removal of material takes place below or above the ablation threshold, which is the minimum laser intensity required to remove material. Below this threshold, most of the laser energy is converted into thermal energy and little material is ejected from the target.

The frequency range dividing thermal and non-thermal effects is between 10^12^ Hz and 10^13^ Hz, corresponding to typical phonon frequencies where thermal effects still play a role. Thus, thermal effects (hot ablation) are significantly more prominent at pulse lengths above 1 ps. As the pulse length increases to 100 ns and above, the ablation will be driven primarily by thermal effects, and expulsion of the melt will dominate. Conversely, non-thermal processes (cold ablation) such as electron heating will be favoured at pulse lengths below 100 fs. Nonetheless, despite the short pulse length, some thermal evaporation can still be observed.

The pulse length also determines the subsequent interaction time of the initially removed material with the laser light. For a pulse length shorter than approximately 10 ps, photophysical and photochemical processes will be limited, as the interaction time is comparable with the time scale for the initial plume formation. Therefore, the number of ions/electrons created due to photodissociation will be small. For femtosecond pulse durations, the typical time scales of electron dynamics are similar to that of the pulse duration. The excitation of solid species by femtosecond pulses is well approximated by the density of electrons generated in the conduction band, while the phonon system remains cold (Fig. 7(b)). The removal of material proceeds *via* a phase (Coulomb) explosion due to overheating of the irradiated material. Conversely, dielectrics require much longer timescales, in the nanosecond range, to undergo the necessary processes (*e.g.*, modification of the index of refraction, damage by cracking, and ablation). In nanosecond ablation, the time window is large enough to dissociate the solid into mostly monoatomic species in the plume. This dissociation is favourable for the controlled deposition of films with a complex composition.

#### 3.1.3. Spot size

In order to achieve an energy density at the target surface, which is sufficiently high to induce plasma formation, the laser must be focused to a small spot on the target. The shape of the focused beam is generally either square or rectangular, with an approximate area of a few square millimetres. The focusing of the beam enables energy density values exceeding a few Joules per square centimetre, significantly above the ablation threshold for many materials.

The area of the target ablated by the laser, commonly referred to as spot size, correlates to the number of removed species per pulse. However, the number of removed species will additionally depend on target properties, for example, the density, absorption of the laser wavelength or the areal energy density. The projection of a laser beam onto the target is achieved *via* two possible approaches. Either the entire beam is focused directly onto the target, or alternatively, an aperture is used to define the beam profile, which is consequently focused onto a target. The disadvantage of the latter approach is that a long imaging beam path (approximately 5 m) is required to obtain a small beam spot; however, the control over the energy distribution and fluence (the energy per unit area) homogeneity is significantly better than the direct focusing of the laser beam. For a 248 nm excimer laser with 20 ns pulse length, a typical material extraction depth for an oxide target is roughly between 10 nm and 20 nm. The number of species removed by such a single pulse is estimated at between 10^21^ m^−3^ and 10^22^ m^−3^, which corresponds to the number of atoms equivalent to a static pressure in the mbar range. The consequence of such a high local density is the drastic reduction of the mean free path (*λ* ≪ mm), meaning that the initial plume expansion is independent of the global background pressure.

**Fig. 8:**
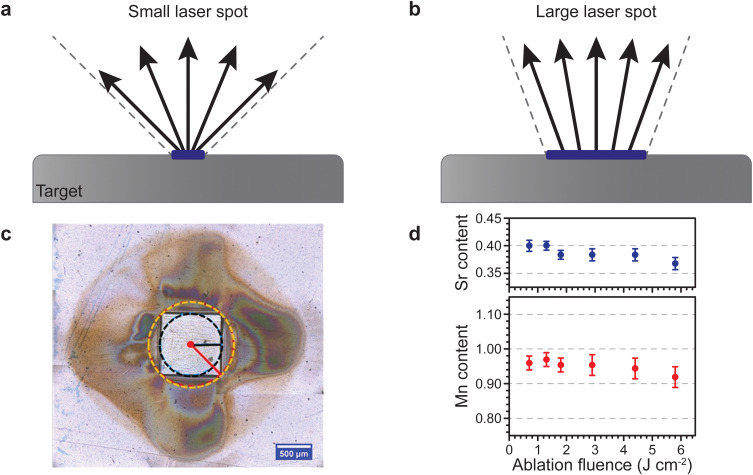
Schematic showing the changes in the opening angle of the plasma plume based on the spot size dimensions for (a) a small laser spot and (b) a large laser spot. (c) Room temperature ambient ablation of polyimide using a square beam profile. The recondensed ablation fragments show the effect of the longest and shortest axes on the material distribution associated with the flip-over effect. (d) The Sr and Mn content as a function of ablation fluence in La_0.6_Sr_*x*_Mn_*y*_O_3−*δ*_ thin films deposited on a (001) Si substrate using an O_2_ partial pressure of 1.5 × 10^−1^ mbar. Panel (c) reproduced from ref. 26 with permission from ETH Zürich, copyright 2016. Panel (d) reproduced from ref. 27 with permission from AIP, copyright 2014.

The aspect ratio of the beam cross section and the size of the beam have a rather peculiar effect on the plume of the ablated material travelling toward the substrate. For one, a smaller spot results in a larger opening angle for the ejected species, and hence such a plasma plume has a large visual appearance. Consequently, a larger spot results in a more tight plume due to the emission of more species and intraplume collisions (Fig. 8(a)). This is similar to the distinction between a point source and a surface source for thermal evaporation. Therefore, a decrease in spot size results in a stronger broadening of the thickness profiles for monoelemental targets, and a directional inhomogeneity of the composition for multielemental targets.

Considering the influences of the beam size on the ablation plume (Fig. 8), it becomes apparent that a rectangle-shaped spot will produce a wider plume profile for the shorter dimension of the spot (Fig. 8(a)), and *vice versa* for the longer dimension (Fig. 8(b)). This is called the flip-over effect. The flip-over effect can even be observed for a square-shaped spot, whereby the plume will preferentially expand perpendicular to the edges, rather than in all directions (Fig. 8(c)).

Furthermore, the composition and propagation of the species in the plume is significantly affected by the size and aspect ratio of the laser beam profile. For example, Fig. 8(d) shows the fluence (areal energy density) dependence on the composition of Sr and Mn in a La_0.6_Sr_0.4_MnO_3_ film grown at an oxygen partial pressure of 1.5 × 10^−1^ mbar. Due to the removal of more species per pulse at an increasing fluence, more scattering events take place in the plume during the time of flight, which results in a deficiency of both Sr and Mn.

### 3.2. Gaseous environment

Collisions of ablated species take place within the plasma plume during the time of flight from the target to the substrate. Such collisions occur at the contact front between the plume and the ambient gas, and near the substrate surface. A background gas can be used during the deposition, not only to tailor the composition of the film, but also to moderate the kinetic energy of the arriving species.^28^ Several parameters affect the extent of collisions and chemical reactions of species at the time of flight, including the pressure and the chemical reactivity of background gas.^28^ This section provides an in-depth discussion of the influences these parameters have on the resultant films.

#### 3.2.1. Atmosphere

The addition of a background gas has multiple effects during the deposition. Importantly, the background gas slows the ablated species, resulting in a reduction of ions ejected from the target. The background gas changes the kinetic energies of the arriving species to the substrate, and can be employed to incorporate elements such as O, C or N to create oxide, carbide or nitride thin films, respectively. Thus, the type of background gas and its pressure influences the crystallinity, thickness and composition of a thin film.

Background gas composition is often tailored to the application. For fundamental studies, an inert gas such as Ar is used to study the kinetic interaction the plasma plume and the background gas. This inert atmosphere can influence the transport of ablated material, the attenuation of the laser light, and the development of surface instabilities. Due to collisions with gas-phase molecules, these effects become more pronounced with increasing gas pressure.^29^ Reactive background gases (*i.e.*, O_2_ or N_2_O) can be used to promote the incorporation of elements into the films, which are otherwise deficient.^30^

When choosing a background gas, it is crucial to consider the type of gas, its mass, the size of its constituent elements species, its reactivity, and mainly, the chosen pressure and its relation to the target-to-substrate distance.

#### 3.2.2. Pressure

The pressure inside the vacuum chamber has direct influence over the number of collisions experienced by the species arriving at the substrate. If an inert gas is used (Ar), the collisions act to control the kinetic energy of the species, thereby tuning the growth process. In the presence of a reactive gas, the partial pressure influences both the kinetic energy of the plasma and, importantly, the chemical composition, due to interactions with the background gas during time of flight.

As the pressure in the vacuum chamber is increased, three distinct pressure regimes are defined: (1) the vacuum-like low pressure regime with minimal interactions between the plume and the background gas; (2) the transition regime corresponding to plume splitting and shock-wave formation, resulting in increasing interactions between the plume and the background gas; and (3) the diffusion-like regime, corresponding to the diffusion of the ablated species away from the plume.^31^

In the vacuum-like regime, the plume constituents expand adiabatically after the termination of the laser pulse. With a decreasing density further from the target, the plume transitions to a free molecular flow. Thus, the propagation of the ablated species and the properties of the resultant films are not affected by the background gas. However, intraplume collisions can still occur, and the species can rebound from the substrate and affect the expansion of the plasma plume at such high kinetic energies (above 1 keV).^28,30^

In this pressure regime, most of the ablated species are concentrated at the centre of the plasma plume. The film thickness is therefore larger in close proximity to the plume expansion axis. The film composition is often similar to the centre of the plasma plume, although is not necessarily the same as the target composition. For multi-element materials, the different species, as well as ions and neutrals of the same element, travel with very high but dissimilar velocities. The consequence of this is the heterogeneity in arrival times, which can influence the growth of the film.^28^

The transition regime generally represents the pressure range between 5 × 10^−3^ mbar and 5 × 10^−2^ mbar. Unlike ablation in vacuum, some interaction between the plume species and the background gas can be expected. The scattering and reactions with the background gas attenuate the plume energy. While chemical interactions between the plasma plume and the background gas species are initiated in this regime, the kinetic energy of the arriving species is still relatively large. Thus, the centre of the plasma plume results in films that are often deficient in the lighter elements of the target material. However, the transition regime allows for the use of reactive background gases, which facilitate the incorporation of O or N species in a film as a function of the pressure and type of background gas.^28^

At pressures corresponding to the diffusion-like regime (between approximately 10^−2^ mbar and 1 mbar), ablated species diffuse away from the plume. The collisions with the background gas reduce the kinetic energy of the species within the plume, such that all species travel at a similar velocity. Thus, films produced in this regime normally present a homogeneous thickness and composition throughout the entire angular range, although it is not necessarily the same as the original target composition.

Pressures beyond the diffusion-like regime (>1 mbar) reduce the mean free path to the extent that structures form before reaching the substrate. As a consequence, the fragments that arrive on the substrate surface create highly porous structures. As one example, ultra-low density films have been demonstrated through the ablation of a pyrolytic graphite target at varying Ar partial pressures, with density values reducing by two orders of magnitude to reach 10 mg cm^−3^ at 10 mbar.^32^

The choice of pressure range can also have an effect on the cation stoichiometry of the films. At low pressures, elements can evaporate from the substrate surface, or desorb from the surface when sputtered by the species arriving with high kinetic energies. At high pressures, elements can also evaporate from the surface, with lighter elements more easily scattered from the surface of the film.

### 3.3. Substrate

**Fig. 9:**
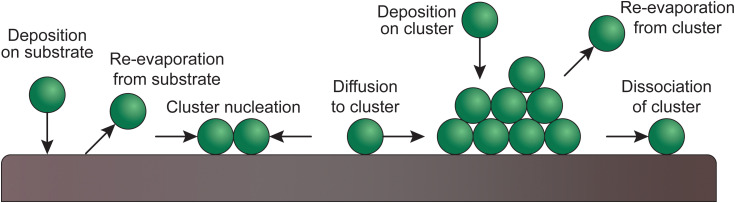
Schematic of atomic processes in the nucleation of clusters of deposited films on a substrate surface.

Upon the arrival of the species on the substrate, the atoms diffuse and react, both with each other and with the substrate. Ordered monolayers can form only if the surface diffusion is high, meaning that the structure or morphology of the resultant thin film is determined by the way the particles nucleate. The substrate plays an important role in this, based on the compatibility of the chemical species and the lattice parameters.^30,33–36^Fig. 9 shows the possible atomic processes in the nucleation of clusters during deposition on a substrate surface.

Typically, crystalline thin films are grown at high temperatures. A high substrate temperature facilitates epitaxial growth by stimulating desorption of impurities, enhancing surface diffusion of adatoms into equilibrium sites and promoting island coalescence. Ensuring the correct substrate temperature is essential and is determined by the thermal contact between the substrate and the holder. Clamps, or metallic (*e.g.*, Pt or Ag) adhesive pastes, are typically used to maintain such contact. Based on the deposition conditions and the sample, the user must find the suitable way to maintain a proper thermal contact and it should be noted that this contact can change while the substrate holder is heating. The actual temperature at the surface of the substrate can be monitored *via* a laser pyrometer, taking into account the emissivity for a given material. The consequences on the film growth and crystallinity are quite important. The location at which the temperature is measured is critical, as differences up to 100 °C can be observed within a distance of a few millimetres. The heater geometry also contributes to the modulation of the plasma dynamics. A hot zone situated at the surface of the substrate can create a local atmosphere of reactive gas, modifying kinetic energies of the incoming plasma species.^26,37^ When the substrate is used to determine the temperature, the emissivity can change during the deposition process. These changes occur due to the deposition of a film with differing emissivity to that of the substrate, and are dependent on the thickness and/or morphology.

The choice of substrate is generally governed by the film to be deposited. The substrate material and the crystallographic orientation of the surface set the lattice parameter observed by the arriving species. This can either induce strain or cause defects that enable relaxation of the film. The surface stability of the substrate and the chemical compatibility with the film material must also be considered. In this section, we discuss these parameters in greater detail.

#### 3.3.1. Lattice parameter and strain

**Fig. 10:**
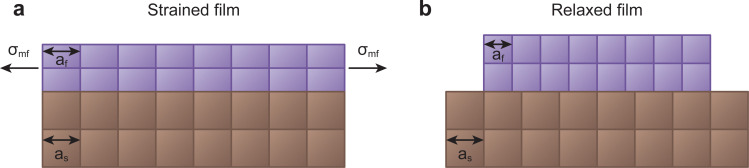
Schematic demonstrating two of the possible growth types for films with lattice parameter different to that of the substrate. (a) Coherent growth in a strained state, whereby the in-plane lattice parameter matches that of the substrate, and (b) incoherent growth in a relaxed state, whereby the film retains its bulk lattice parameter.

The possibility for epitaxial growth is first determined by the lattice mismatch (*ε*_m_). The mismatch can be calculated using the lattice parameters of the substrate surface (dependent on the crystallographic orientation) and the film, as shown in eqn (6).6
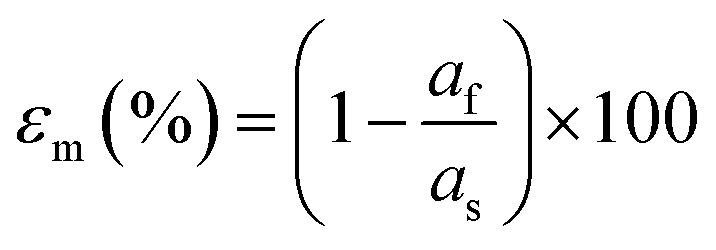
Here, *a*_f_ and *a*_s_ correspond to the lattice plane spacing in the in-plane direction for the film and substrate, respectively.^26,37^ In many cases, approaching 1% strain can be a major challenge. For a small lattice mismatch, the interfacial energy is minimal, which is more likely to form coherent epitaxy (Fig. 10(a)). An expanded discussion on the meaning of coherence is presented in the following section. In this case, it is possible for the film growth to proceed with a one-to-one match of the lattice planes, up to a critical thickness where it becomes energetically favourable for the film to accommodate dislocations. Such dislocations are generated at the film surface and migrate towards the interface.^38,39^

In some cases, relatively large values of lattice mismatch (up to a 7%) can be accommodated at the interface.^40^ Above this value, the film commonly grows in a relaxed state, either textured or largely polycrystalline (Fig. 10(b)).^41^ However, in large lattice mismatch systems, epitaxial growth of thin films is still possible, as explained by the domain matching epitaxy. If the film and the substrate have similar crystal structures, the matching of planes can become equivalent to the matching of lattice constants.^42^

The unit cell structure of the film prefers to take on the intrinsic lattice parameter of its relaxed form, as this is the energetically favourable configuration in the bulk. During the formation of the first monolayer of the film, the arriving species attempt to match the lattice parameter of the substrate. The film will take on the lattice parameter of the substrate in case of sufficient energy of the arriving species, suitable substrate temperature, small lattice mismatch, and compatible chemical species. This will exert a large misfit stress (*σ*_mf_) on both the film and the substrate, altering the lattice parameter of the film in all crystallographic directions in an attempt to preserve the unit cell volume. If the film experiences a tensile in-plane strain, thereby increasing the in-plane lattice parameter, the out of plane lattice parameter will reduce, and *vice versa*. The ratio of this change between the in-plane and the out of plane lattice parameters is called the Poisson ratio, linked directly to the elastic properties of the material. Notably, this relation is generally exclusive to sufficiently thin films, resulting in strain relaxation over larger thicknesses due to the elasticity of the film.^42,43^

**Fig. 11:**
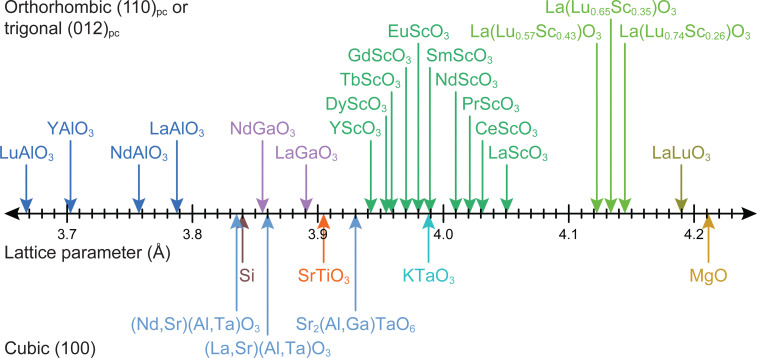
The lattice parameters for selected single-crystal substrate materials employed for oxide thin film growth by PLD.

Strain engineering allows for the controlled tuning of material properties, achieved using a range of different commercially available single crystalline substrates (Fig. 11), covering the lattice parameter range between 3.68 Å (LuAlO_3_) and 4.21 Å (MgO). By choosing the lattice parameter of the substrate and exerting a predefined strain on the film, new functionalities can be unlocked. An excellent example is the quantum paraelectric SrTiO_3_, which possesses a cubic space group, exhibits polarisation and therefore ferroelectric properties when strained.^44^ As misfit dislocations reduce the strain in the film, one has to be particularly careful to sufficiently optimise the parameter space for the deposition such that the prevalence of defects is reduced to a minimum. This can be complemented by the *in situ* measurement techniques discussed further in Section 4.

#### 3.3.2. Coherent, incoherent and semi-coherent growth

**Fig. 12:**
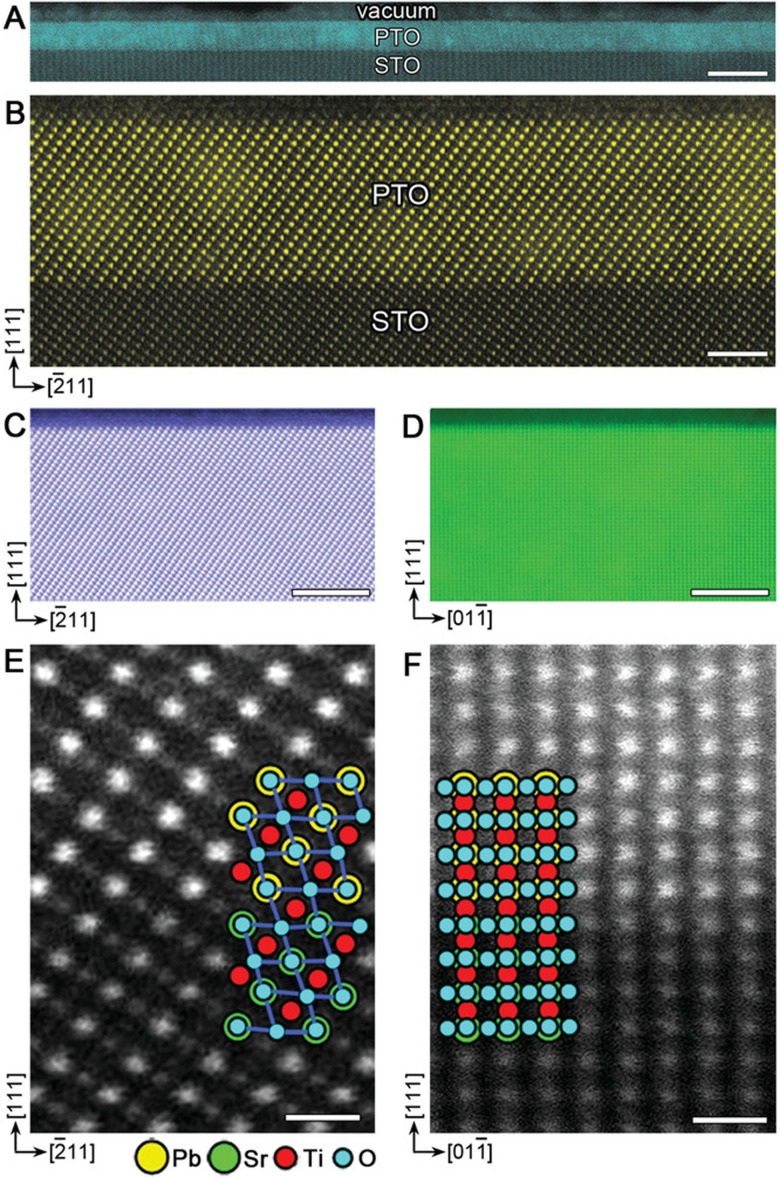
HAADF-STEM images of coherently grown PbTiO_3_ films on SrTiO_3_ (111). (a) Low- and (b) high-magnification images of a 6 nm film viewed along 〈110〉 SrTiO_3_. (c), (d) surface structures and (e), (f) magnified interface structures of a 15 nm film viewed along 〈110〉 and 〈112〉, respectively. The atomic structures are shown as two insets in (e) and (f), where the yellow circles correspond to Pb^2+^ columns (superposed with O^2−^ anions, blue circles); green and red circles denote Sr^2+^ (superposed with O^2−^) and Ti^4+^ columns, respectively. Pure O^2−^ columns contribute negligible image intensity due to their small Z. Scale bars: (a) 10 nm; (b) 2 nm; (c), (d) 5 nm; (e), (f) 0.5 nm. Reproduced from ref. 45 with permission from Wiley, copyright 2019.

**Fig. 13:**
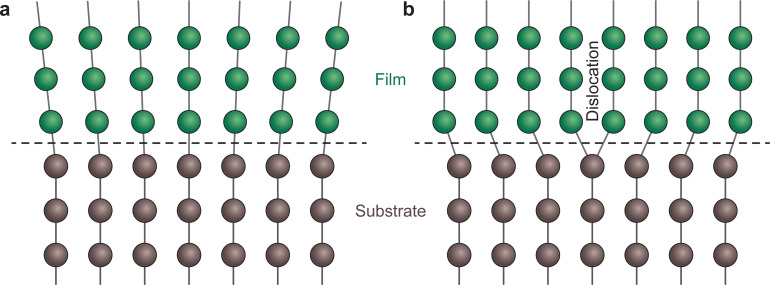
Schematic representation of an interface (dashed line) for (a) a coherent boundary and (b) a semi-coherent boundary exhibiting misfit dislocations.

Thin film growth includes the formation of interfaces, for example between the film and substrate, underlying or overlying films, and the ambient environment. The quality of interfaces can impact the functional properties of the film; thus, it is important to understand the influence of the possible growth types. A coherent interface is formed when the two interfaced crystals possess a matched atomic configuration in both phases, and the two lattices are continuous across the interface. An example of this is shown in the high angle annular dark field (HAADF) scanning transmission electron microscope (STEM) images of PbTiO_3_ ultrathin films grown by PLD on SrTiO_3_ (111) substrates in Fig. 12.^45^ Coherent interfaces can be achieved in material pairs with mismatched lattice spacing by applying in-plane strain on one or both lattices (Fig. 13(a)). However, such strain increases the energy of the system. This means that for a moderate misfit strain, it is energetically favourable to form semi-coherent interfaces. Here, the mismatch is periodically alleviated through misfit dislocations (Fig. 13(b)). As the misfit strain increases between two material pairs, increasing the interface energy, the spacing between dislocations decreases. This occurs up to the point where the dislocation fields overlap, and the interface becomes incoherent. Incoherent interfaces are similar to low energy and high angle grains, independent of orientation.^39^

#### 3.3.3. Chemical compatibility and surface stability

Selecting a substrate with the highest structural and chemical match with the thin film material significantly contributes to superior surface morphology and lower defect density of the thin film.^46^ An example of such selection is the Bi–Sr–Ca–Cu–O (BSCCO) superconductor. In this material, the interaction of the cation elements between the substituent and the constituent elements is influenced by the type of substrate used.^47^ Another example of the importance of chemical affinity is the growth of thin continuous Au layers on SiO_2_ substrates. The chemical affinity between Au and SiO_2_ is low, thus the growth proceeds in a polycrystalline manner due to the island-type growth. Using two-dimensional MoS_2_ nanosheets as an intermediate layer between SiO_2_ and Au allows for the preparation of thin continuous Au layers as suitable electrical conductors.^48^

**Fig. 14:**
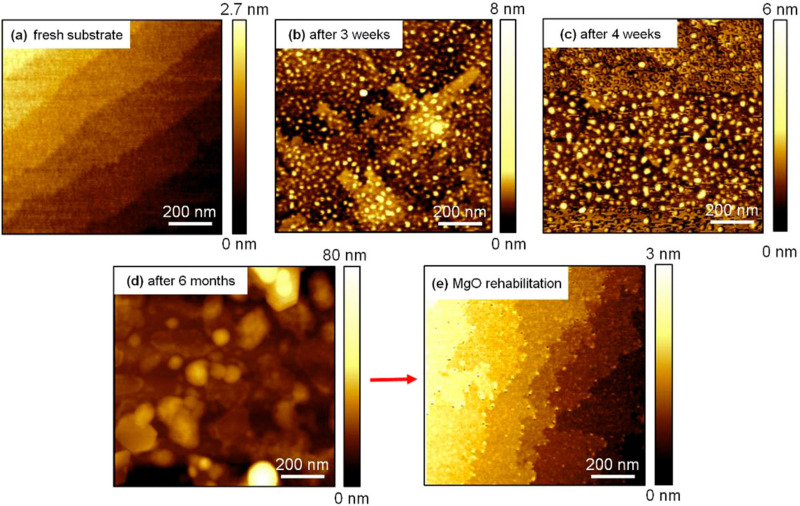
The surface morphology of a MgO (100) single-crystal (a) directly following surface reconstruction *via* annealing, followed by (b) three weeks, (c) four weeks and (d) six months of storage, and (e) after an additional annealing following storage. Reproduced from ref. 50 with permission from AIP, copyright 2018.

Impurities, physical and chemical adsorbates, and planar defects on a substrate surface can immediately interfere with the growth process. Thus, they must be removed or treated before the deposition.^49,50^ To obtain controlled substrate–film interfaces, vicinal substrate surfaces showing a high density of uniform step-terrace features with uniform chemical termination are desired. The terraces possess single unit cell steps arising due to the slight miscut of the substrate relative to the desired crystallographic axis, typically below 0.3°. Surface pre-treatment is essential for coherent growth and has a substantial effect on creating the perfect surface with single chemical termination. In the case of SrTiO_3_, varying the surface pre-treatment methods and conditions can lead to either SrO or TiO_2_ termination.^51^ Another example of the effect and importance of surface pre-treatment is the annealing of MgO surfaces at high temperatures before deposition. Under ambient conditions, chemisorption of small molecules (*e.g.*, H_2_O and CO_2_) occurs on the MgO, thus the surface reactivity and stability are a concern for extended storage of the substrates (Fig. 14).^52^ The hygroscopic nature of MgO further deteriorates the quality of the surface, interface, and crystallinity of the deposited films, thus it is recommended to store such air-sensitive substrates in inert environments (*e.g.*, Ar) and pre-treat the surface directly before deposition. Annealing at high temperatures increases the homogenisation of the substrate surface by enabling the reorganisation of the surface atoms, which in turn enhances the stoichiometry control, texture and nanostrain.^53^

#### 3.3.4. Orientation

In a crystalline film, each constituent grain has a specific crystallographic orientation, relative to a fixed reference direction. When the distribution of grain orientations is not random, the film exhibits a preferred crystallographic orientation.^54^ Assuming that the grains with a particular crystallographic orientation dominate the evolving structure, the orientation preference of the thin film is dominated by two factors:^55^

• The crystal nuclei are shaped in a geometry that results in minimum free surface energy; and

• All crystal nuclei have a similar shape.

During the coalescence and nucleation of atomic species on the surface of the target, the nuclei matching the direction of the substrate consume the non-matching nuclei and determine the preferential orientation of the later stages of film growth. Notably, the matched direction of the substrate is not necessarily the same crystallographic orientation, rather the energy-minimised direction of the film relative to the substrate face. This means that, as an example, a (001) oriented film can grow on a (110) oriented substrate, provided the conditions such as the substrate temperature, gaseous environment and partial pressure are favourable.^46,56^

The control over the crystallographic orientation of a growing film can be highly important for many technological applications, in particular ones that exploit anisotropic properties of materials. Along different crystallographic axes, significant differences in charge extraction and epitaxial growth have been reported for different materials. Efficient charge separation in photocatalysts has been demonstrated to be dependent on the crystalline facets, using BiVO_4_ as a model material. The reduction reaction with photogenerated electrons and oxidation with photogenerated holes takes place on the {010} and {110} facets.^57^ For rutile TiO_2_ in different surface orientations, the photocatalytic degradation activity for organics follows the order (101) > (110) > (001) > (100).^58^ Similarly, the increased activity of certain photochemically reactive sites in SrTiO_3_ has been attributed to preferential diffusion of excitons along selected crystallographic orientations.^59^ In ferroelectric thin films, the polarisation direction is set along crystallographic orientations, governed by the direction of spatial atomic displacement of ions. Thus, crystallographic growth direction of the film is of high importance to technological applications. The orientation dependence of ferroelectric properties has been shown in La-doped Bi_4_Ti_3_O_12_ ferroelectric thin films. The (104) orientation of the films exhibits approximately 1.5 times higher remanent polarisation than the (118) orientation, while a marginal polarisation magnitude is observed in the (001) oriented films.^60^

### 3.4. Target

One of the major advantages of PLD is the ability to transfer complex stoichiometries from a target to the substrate. Thus, the target forms the basis for the stoichiometry of the deposited thin films. However, as discussed in Section 2, the transfer of desired compositions is a challenge, arising from the intrinsic properties of the material and its constituent atoms. Additionally, the physical structure of the target itself can play an important role in producing high-quality thin films. This section will discuss the common pitfalls regarding the target preparation and handling, and strategies to achieve the desired stoichiometry in the resulting films.

#### 3.4.1. Material and composition

**Fig. 15:**
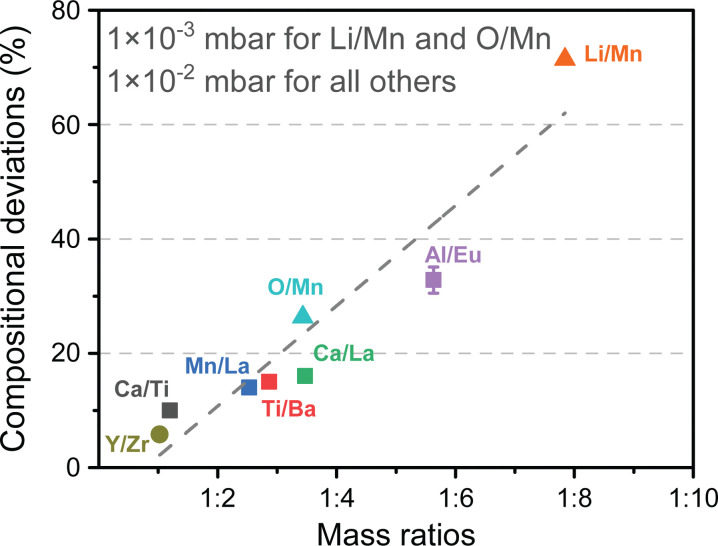
The observed compositional deviation for multielemental oxide thin films possessing mass differences between cationic species. As the mass ratio of the constituents increases, higher deviations from stoichiometric compositions are observed. Reproduced from ref. 26 with permission from ETH Zürich, copyright 2016.

The compositional transfer between the target material and the substrate is an important factor to consider for a deposition. The composition of the thin film can deviate up to 30% from that of a target. If the atoms comprising the target materials have a significant differential in the mass, the lighter elements tend to be accelerated faster and scatter further away from the target-to-substrate direction in comparison to the heavier elements. This results in the lighter species arriving at the substrate surface first, albeit with a lower relative proportion as a consequence of scattering. This effect is hypothesised to cause significant compositional deviation.^61^ For instance, light elements such as Li are distributed over a larger volume in the plasma in comparison to the heavier elements. Li is also easily removed from the substrate due to sputtering from the high energy incident species or evaporation.^62^ The effect of the compositional deviation in a film as a function of the mass ratio (Fig. 15) highlights this issue, emphasising the difficulty in obtaining the correct film composition when combining light and heavy elements.^26^

Similarly, differences in the volatility of the species comprising the target will influence the film stoichiometry. An increased volatility results in the preferential melting and desorption of the material from the target during ablation, and re-evaporation from the substrate after arrival. Often, the resulting films will be deficient in the light and/or volatile elements, which can alter the structural or the functional properties of the film. The target can be prepared with an excess of the volatile atomic species to reduce the extent of these effects. However, this measure would have limited impact on light elements. In this case, the deposition parameters must be tailored to reduce the frequency of scattering events (*e.g.*, reducing the pressure or increasing the spot size) while maintaining a consistent kinetic energy (*e.g.*, reducing the fluence).

The absorption properties of the target material must also be considered. These properties are generally guided by the band gap of the target material, therefore requiring ablation by a laser wavelength with an energy above this value. The ablation of a target with a laser wavelength below the band gap of the material would result in reduced ablation rates, which can be compensated by increasing the laser fluence. On the other hand, the intrinsic thermal properties of the target material can lead to surface segregation as the fluence is increased. In the case of metallic targets, droplets may form and therefore degrade the quality of the resulting thin film.^63^ Considering the deposition of multi-element thin films, a combination of these effects is likely to occur during deposition. The composition of the target can change throughout ablation due to differences in the melting and vaporisation points of the material, consequently altering the composition of the resulting thin film. Similarly, rebounding of the species from the substrate can redeposit on the surface of the target, altering the surface composition, which in turn redeposits on the substrate.^61^

#### 3.4.2. Surface quality

The target density and mechanical stability can influence particulate formation. An example of such influence is observed in yttria-stabilised zirconia (YSZ) thin films, where either the cubic or the tetragonal phase can form, depending on the density of the target.^64^ Similarly, higher density targets yield films with reduced roughness in the case of TiO_2_ targets.^65^ The common density of a material used for laser ablation in a PLD system should be above 90%. Failing to reach such a value will impact the deposition rate and can increase the ejection of macroparticles onto the substrate.

The degradation of the target surface during ablation can have a significant impact on the quality and composition of the thin film. The target material ejects a mixture of electrons, ions, neutrals, molecules and clusters, as well as undesirable particulates, all of which are incorporated in the deposited film. Thus, it is critical to ensure a clean surface of the target before ablation, commonly achieved through mechanical polishing (*i.e.*, sanding) and *in situ* pre-ablation of the surface. Furthermore, the directional particulate formation, caused by an approximately 45° incidence angle of the laser, can be partially mitigated through bidirectional ablation.^66^

The importance of target storage must be highlighted. Certain materials may undergo reactions upon exposure to air. These reactions can cause the incorporation of unintended atomic species and/or segregation of certain atoms on the surface of the target, resulting in changes to the optical properties. Thus, it is important to ensure proper storage of the targets, and to pre-ablate the surface of the target before the deposition.

#### 3.4.3. Target to substrate distance

During a PLD deposition, most of the ablated material is contained within a ±30° angular range of the plume. Thus, the target to substrate distance has an evident impact on the angle of the expansion plume, and a direct impact on the film thickness and composition. A larger target to substrate distance results in lower film thickness, while a smaller distance results in the strong rebound of species due to high kinetic energies. In terms of the elemental composition of thin films (discussed in Section 3.4.1), a distance below the mean free path can be critical to element distribution, due to the interactions between the plume species and the background gas. Such interactions are required during depositions with a reactive gas, such as O_2_, O_3_, N_2_O, N_2_, or NH_3_.^26^

## 4. *In situ* monitoring techniques

The importance of understanding the individual parameters relating to thin film growth in PLD has been described throughout this tutorial, in Sections 2 and 3. Indeed, one can precisely control a wide range of parameters, which can mean the difference between a polycrystalline and rough film, and one that is grown coherently and epitaxially, resembling a single crystal. In this part of the tutorial, we build on these concepts by discussing selected methods used to follow the growth of materials *in situ*. This analysis provides an additional dimension to the understanding of the growth mechanism, and in certain instances, the evolution of the functional properties. The operating principles of four commonly used *in situ* techniques are described, and the information they can provide is discussed in this section.

### 4.1. Reflection high-energy electron diffraction

One of the most common techniques used in conjunction with PLD is reflection high-energy electron diffraction (RHEED). The ubiquity of this accessory is due to its capability for providing real-time information about the growth mechanism, and if following a two-dimensional growth mechanism, the surface reconstruction process.^67^

**Fig. 16:**
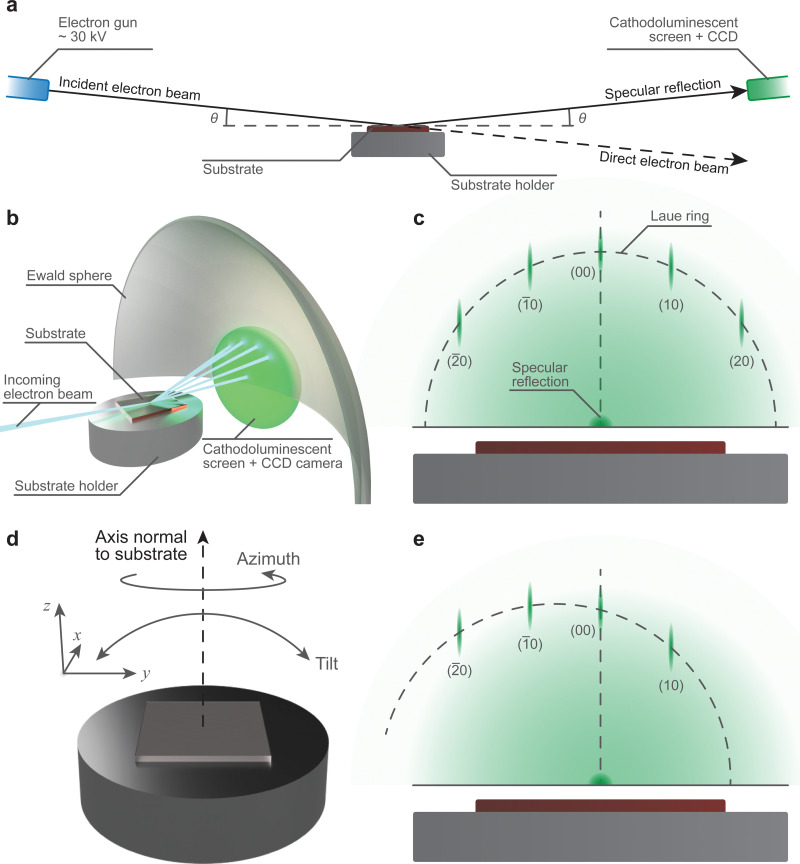
Schematic demonstrating the required instrumentation and the geometrical layout of the RHEED technique. Schematic showing (a) the layout of the RHEED, including the Ewald sphere and the diffracted electron beam, and (b) the resultant diffraction spacing on the cathodoluminescent screen, with the Laue ring trace. The representative RHEED pattern is shown together with the Laue ring for (c) an electron beam aligned with a crystallographic direction of the substrate, (d) demonstration of the types of substrate holder movements used to align the substrate, and (e) the pattern for a misaligned electron beam.

The necessary instrumentation (Fig. 16(a) and (b)) consists of an electron gun powered by a high voltage power supply, a cathodoluminescent screen (alternatively referred to as a phosphor screen), and a charge-coupled device (CCD) camera. The combination of the cathodoluminescent screen and CCD camera provides a simple method to visualise the diffraction pattern from the substrate (Fig. 16(c)) in low pressure conditions and perform real-time quantitative analysis on the intensity of the diffracted spots. In order to convert the impinging electrons into visible light (*i.e.*, cathodoluminescence) in vacuum chambers, glass windows coated with approximately 100 μm of ZnS are used. This coating emits light at 450 nm, in the green region of the visible light spectrum. The incident electron beam is generated using an electron gun. To obtain an electron beam with sufficiently high kinetic energy, the gun is driven at a potential difference of approximately 30 kV. The electron optics, similar to those used in scanning electron microscopy and transmission electron microscopy, focus the beam onto the surface of the substrate. It is necessary for the incident electron beam to be oriented at grazing incidence (Fig. 16(a) and (b)) with respect to the substrate, with the angle *θ* between 0.5° and 4°. The coupling between the high kinetic energy and the low incoming angle of the incident electron beam is the primary reason for the informative nature of RHEED: this combination results in probing the topmost layers of the substrate through elastic collisions of the electron beam.

The diffraction arising in RHEED is highly sensitive to the geometrical layout of the instrumentation. Thus, it is important to ensure the angles of the incident electron beam and the specular reflection are equal. This can be achieved during the commissioning stage with a fixed sample holder, although deviations may occur during the lifetime of the chamber. The desirable alternative is to use a sample holder with variable tilt angle and *z*-axis translation (Fig. 16(d)), which is capable of correcting the drift. Additionally, RHEED is sensitive to the crystallographic direction of the substrate, requiring adjustments in the rotation of the substrate to align it relative to the electron beam (Fig. 16(e)). To make the necessary corrections, two options may be used: (1) the coarse adjustment is carried out through correcting the azimuth angle of the substrate, and (2) the subsequent fine adjustment can be performed by regulating the beam direction through the electron gun optics. Thus, the control over the substrate position is immensely beneficial in the preparation for RHEED measurements.

The theoretical foundation for the method of operation in RHEED, and indeed the requirement to use the instrument to its full potential, is beyond the scope of this tutorial series due to its complexity. As such, we will omit the majority of this theoretical basis in this tutorial and direct the readers to the works of Bland and Heinrich,^68^ Braun,^69^ and Wang and Lu^70^ in order to better understand the fundamental principles of the technique. In RHEED, four key pieces of information can be obtained *in situ*:

• The surface quality of the substrate.

• The crystallographic orientation of the film relative to the substrate.

• The growth mode of the film; and

• The number of atomic layers deposited when following a layer-by-layer growth mode.

The former two have been discussed in depth, including the relevant background, in the sources provided above; therefore, we will focus on the latter two points.

**Fig. 17:**
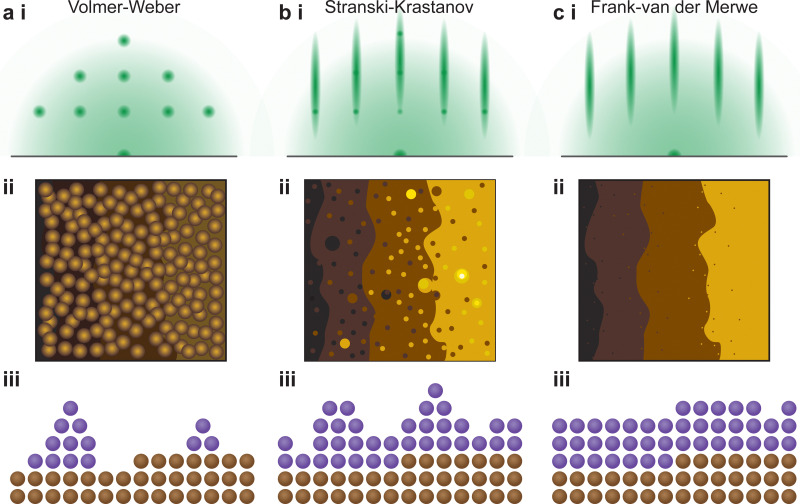
Schematic representation of the identifying features of the three thin film growth modes, (a) Volmer–Weber, (b) Stranski–Krastanov and (c) Frank–van der Merwe, showing the representative (i) RHEED patterns, (ii) atomic force microscopy height traces and (iii) cross-sectional distribution of atomic species on an atomically flat substrate.

The understanding of the growth mode is critical for the growth of coherent epitaxial thin films. The growth of a thin film can proceed through one of three primary modes, island (Volmer–Weber, Fig. 17(a)), layer and island (Stranski–Krastanov, Fig. 17(b)) or layer-by-layer (Frank–van der Merwe, Fig. 17(c)) growth mode, as discussed in Section 2.4.2. The type of growth observed for a particular material and substrate combination is primarily dependent on the atomic surface energies of the substrate and the film. However, as discussed in the previous sections, a range of parameters can further influence the growth mode. In order to improve the understanding of thin film growth, RHEED can be utilised for the monitoring of the growth mode, as well as dynamic changes in the growth mode, in real time throughout the deposition.

In particular, the observation of the RHEED pattern as a function of time can provide insights to this mechanism. The three-dimensional (island) growth mode results in distinct diffraction spots as part of the RHEED pattern (Fig. 17(a)-i), which result from the electron beam passing directly through the three-dimensional structures (Fig. 17(a)-ii, iii). Following this type of growth, the resultant morphology is non-homogeneous and therefore the terrace steps of the substrate are either difficult to discern or are not visible altogether. Conversely, the other two growth modes result in a streaky RHEED pattern. The intermediate growth mode, a combination of layer-by-layer and island growth types (Fig. 17(b)-ii, iii), results in a pattern that consists of streaks corresponding to a flat sample, along with generally discernible diffraction spots (Fig. 17(a)-i). As such, the layer-by-layer growth mode shows only a set of streaks (Fig. 17(c)). During a deposition, transitions between the three fundamental growth modes can occur. These changes are directly observed in real time through changes in the RHEED pattern.

**Fig. 18:**
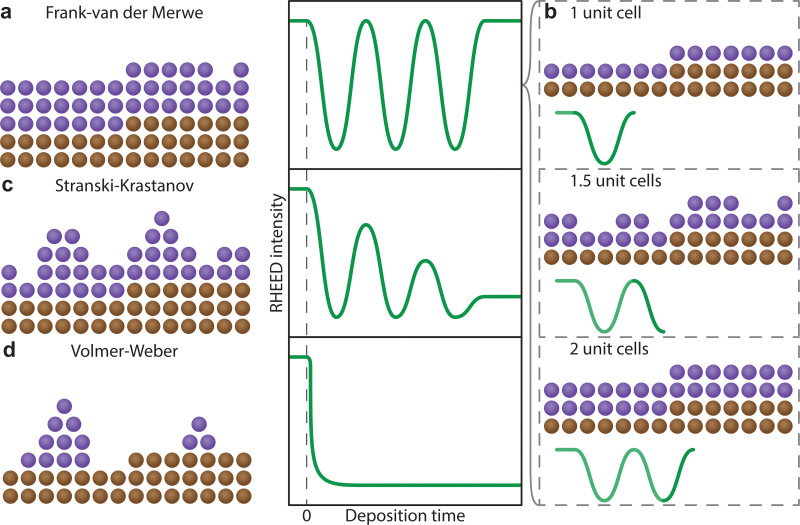
Schematic showing the RHEED intensity profiles as a function of deposition time. (a) Frank–van der Merwe growth, together with (b) the RHEED intensity progression during the growth of a single unit cell, (c) Stranski–Krastanov growth, and (d) Volmer–Weber growth.

In the instance of a layer-by-layer or a layer and island film growth, the thickness of the film can be observed. Due to the cathodoluminescent nature of the screen, the flux of diffracted electrons is converted directly into photon intensity. The photon intensity can be monitored as a function of time during the deposition. In RHEED, a reduction in the intensity of a diffracted spot is attributed to a reduced homogeneity of the morphology, arising from a broadening in the electron scattering angles. As a layer reconstructs at the interface to form an atomically flat surface, the intensity will increase to a maximum. Perfect homoepitaxial growth (that is, both the film and the substrate consist of the same material) can result in intensity returning to the initial maximum, suggesting complete reconstruction of a single layer (Fig. 18(a)). The number of unit cells on the surface can therefore be counted with high precision, as each unit cell corresponds to a single period of oscillation (Fig. 18(b)). For most heteroepitaxial films, the presence of a lattice mismatch (alongside other variables discussed in Section 3) yields a growth corresponding to a combination of layer and island growth modes, manifesting on the intensity graph as damped oscillations (Fig. 18(c)), decreasing in the maximum intensity as a function of the number of deposited layers. In this instance, it becomes increasingly impractical to count the number of layers beyond a particular thickness due to the reduced intensity. Finally, in the case of island growth, the RHEED intensity follows an exponential decay trend as a function of the deposition time (Fig. 18(d)), due to incomplete reconstruction of the surface arising from agglomeration of the adsorbed species. Nonetheless, the real time observation of the RHEED pattern and the intensity profile in real time is a powerful tool towards tailoring the growth parameters of thin films with the aim of coherent and epitaxial growth.

### 4.2. Multi-beam optical stress sensor

In film deposition, it is very common to grow films with composition differing to that of the substrate in order to study properties associated directly with the film. In the case of epitaxial growth, this is known as heteroepitaxy.^34,71,72^ If film and substrate material are identical, this is called homoepitaxial growth.^73,74^ The choice of substrate is critical, as isolating the properties of the film requires a substrate material that will not interfere with the functional properties of the film. The lattice parameters of the desired substrate are not necessarily the same as that of the film (as discussed in Section 3.2), resulting in an induced in-plane strain on the film during the deposition (taking into account the thickness of the substrate, which is generally orders of magnitude larger than the film). Moreover, the stresses experienced by the film even at low strains (*i.e.*, approximately 1%) are capable of inducing properties not observed in bulk films when relaxed. The purposeful induction of strain has expanded tremendously in recent times, forming a separate field of study, termed strain engineering.^75^ Thus, the measurement of the evolution of strain during film growth has become an important aspect in thin film deposition, also for PLD, where the strain state of a film can be detected and monitored *in situ* down to the unit cell level.^76–78^ A change in curvature can also be observed due to oxygen ion mobility, similar to the concept of ion conducting materials. In this case, the curvature is induced due to changes in the oxygen content near the substrate-film interface, rather than the lattice mismatch.^79–81^

**Fig. 19:**
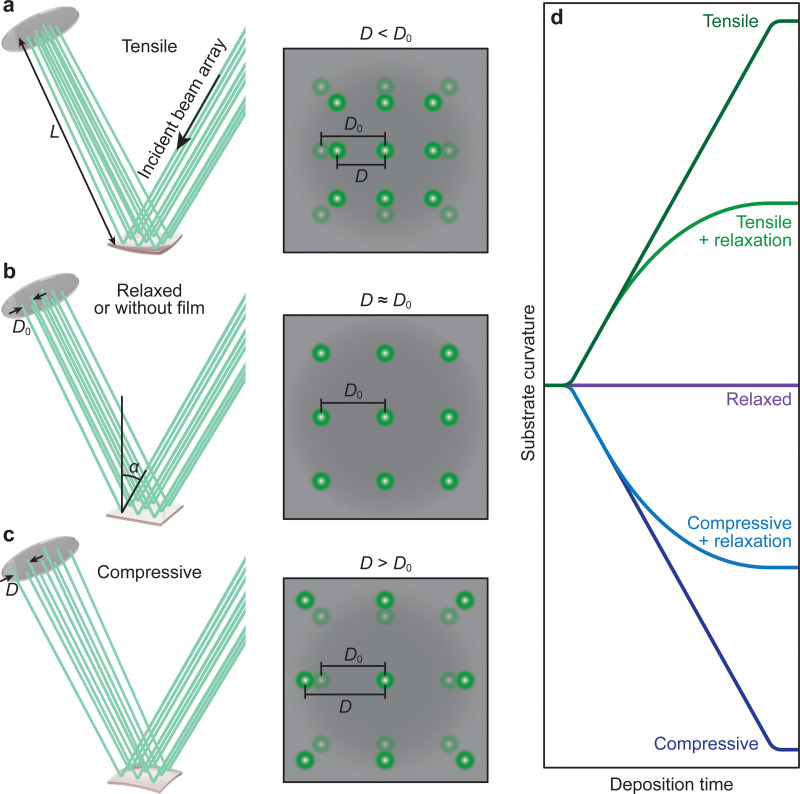
Schematic representation of the MOSS laser array reflecting from the substrate, together with the representative changes in the spot spacing observed on the CCD camera for (a) tensile strain, (b) no strain, and (c) compressive strain. (d) The evolution of the curvature as a function of the deposition time for various cases of strain transfer during film growth.

The strain of a thin film results in a minute curvature of the substrate, which, over subsequently large distances, can be measured using optical techniques.^82–85^ The relationship between the curvature of the substrate and the material properties is given by the Stoney equation, which will be presented and discussed further in this section. This is precisely the method of operation for the multi-beam optical stress sensor (MOSS). MOSS employs a large incidence angle in relation to the substrate surface plane (approximately 60°). A laser beam, which is in the visible wavelength range, is split into multiple parallel beams in both the *x* and *y* direction, resulting in a spatially separated array of laser spots reflecting from the substrate to a detector (Fig. 19). As the film grows on the substrate in a coherent or semi-coherent manner, the stress arising from the difference in the film and substrate lattice parameters results in a curvature of the substrate, and when the MOSS is used, the separation between reflected spots changes, which can be observed optically over a sufficiently large path length. This large path length is necessary, as the curvature (*κ*, represented in *R*^−1^, where *R* is the radius of curvature) can be on the order of several km^−1^. The reflected laser beams are subsequently visualised using a CCD, and the separation between the spot centroids (*D*) can be monitored in both the *x* and *y* directions (following Fig. 16(d)) as a function of deposition time. It becomes clear that MOSS is a semi-quantitative technique, measuring *D* relative to an equilibrated value of the spacing *D*_0_ prior to deposition. Thus, it is important to ensure that during the time *D*_0_ is measured, the temperature and gaseous environment are stable, as these factors can cause the value to fluctuate.

The relative change in MOSS spacing is geometrically correlated with the change in the substrate curvature in response to stress applied by the growing film through eqn (7).7
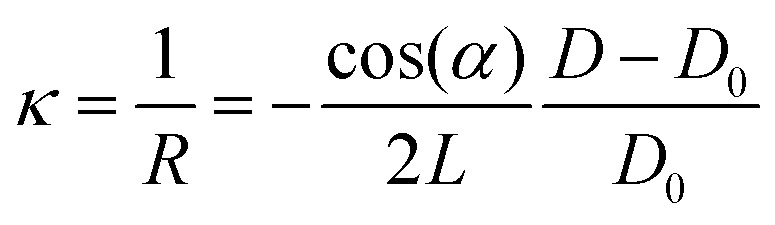
Here, *α* is the angle of reflected light relative to the axis perpendicular to the substrate surface (in the centre), and *L* is the distance from the substrate to the CCD camera (Fig. 19(a)–(c)). From this, it is possible to deduce the direction of the stress (Fig. 19(d)). In the instance of a positive curvature (*i.e.*, a reduction in *D* relative to *D*_0_), the film experiences tensile stress. Conversely, in the case of a negative curvature (*i.e.*, an increase in *D* relative to *D*_0_), the film experiences compressive stress.

The magnitude of the stress can be further quantified if the mechanical properties of the substrate are known, along with the deposition rate of the film. This quantification follows the Stoney equation, which can be adapted to a variety of cases (the review of Janssen *et al.*^86^ provides further clarifications on the information presented herein). The simplest form of the Stoney equation utilises the biaxial modulus (*M*_s_) of the substrate, as shown in eqn (8a).8a
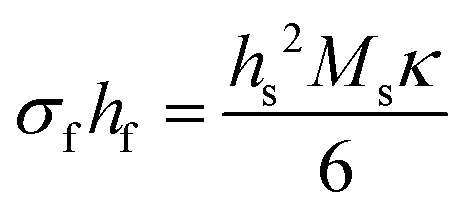


In this form, the left-hand side consists of the stress experienced by the film (*σ*_f_) multiplied by the instantaneous thickness of the film (*h*_f_), and on the right-hand side *h*_s_ corresponds to the substrate thickness. The MOSS data is commonly presented by plotting the stress-thickness product (*i.e.*, *σ*_f_*h*_f_) as a function of deposition time. Knowing the deposition rate, the stress on the film can be quantitatively determined at a particular film thickness value. The biaxial modulus is not commonly reported for the entire vast range of substrates available, thus three alternate forms of the Stoney equation can be employed. The first, shown in eqn (8b), assumes the Young's modulus (*Y*_s_) and the Poisson's ratio (*ν*_s_) of the substrate are known.8b
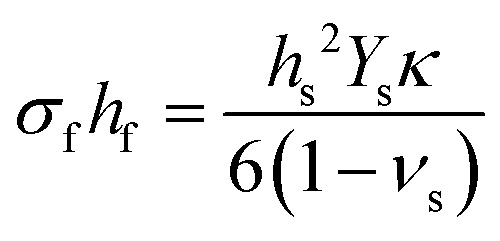


The Young's modulus and Poisson's ratio are values often reported for the majority of substrate materials. Hence, this form of the Stoney equation is most often used. However, in the case of materials with anisotropic properties, or materials for which the former two mechanical properties cannot be found in literature, the elastic coefficients (*c*_*ij*_) or the compliance coefficients (*s*_*ij*_) can be used instead. For a cubic material, the tensor form of Hooke's law reduces from an 81-component tensor to a three-component tensor (*i.e.*, *c*_11_, *c*_12_, and *c*_44_), and thus the Stoney equation can be rewritten as shown in eqn (8c).8c
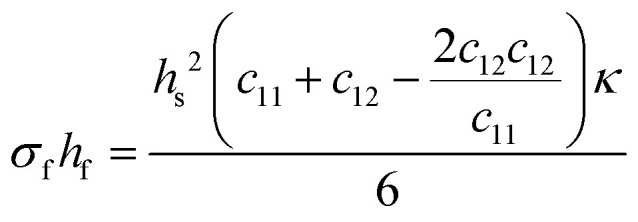


Finally, if the compliance coefficients (*s*_11_ and *s*_12_ for a cubic material) are known, the Stoney equation takes on the form shown in eqn (8d).8d
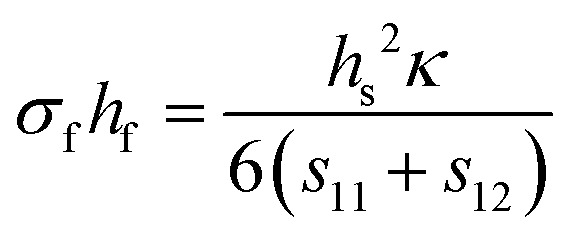


Understanding the stress behaviour of a film during deposition can provide unique insights into the growth of the film. For example, the relaxation of stress can be observed in real time and linked directly to the deposition parameters. Furthermore, the temporal evolution of the film stress can be compared directly to literature values for the purposes of applications in strain engineering.

### 4.3. Mass spectrometry

Generally, a mass spectrometer (MS) measures the mass (or rather the mass-to-charge ratio, *m*/*z*, where *m* corresponds to the mass and *z* corresponds to the charge) of ionised species. For thin film studies, a MS is used to determine the composition of a film or an isotope ratio for depth resolved diffusion studies, called secondary ion MS (SIMS). The MS is coupled with an ion gun and a flood gun, whereby the ion gun is used as the primary ion beam (typical gases are Ar^+^, O_2_^+^, Cs^+^) to remove material in a controlled way to be detected with the MS. The flood gun is used to compensate excess charges of highly insulating samples, which are present due to the removal of material with the ionised primary beam.

When an array of ions impinges onto a surface, it ejects material from the surface of the film through a process called sputtering. Multiple types of sputtering exist (*e.g.*, physical, chemical, external or internal). The former two are important for SIMS. Physical sputtering relies on the kinetic energy of the incident ions to be large enough to overcome binding forces of the film. Conversely, chemical sputtering requires the kinetic energy of the incident beam to be large enough to induce chemical reactions in the film, thereby generating instability in the molecules, resulting in desorption from the surface.^87^ Subsequently, the molecules sputtered from the surface of the film are extracted by the endcap of the MS for analysis.

The sputtering of atoms in the film depends on multiple factors, which are shown in eqn (9). Here, *I*_p_ is the primary particle flux, *y*_m_ the sputter yield, *α*^+^ the probability of ionisation into cations, *θ*_m_ the relative composition of a particular cation in the layer, and *η* the transmission function.

9 *I*^m^_s_ = *I*_p_*y*_m_*α*^+^*θ*_m_*η*

The quantity of ions received by the MS endcap is influenced by factors such as the matrix effect, the surface coverage, the background pressure, the out of plane crystallographic orientation, or the angle of emission of the detected secondary ions. The contributions of these factors vary between differing ions, which leads to difficulties in the quantification of SIMS analysis.

They are two main ways to perform SIMS: surface SIMS or dynamic SIMS. The two methods are differentiated by the ion current density used to bombard the film. Surface SIMS (low ion current densities of approximately 1 μA cm^−2^) sputters the top monolayer away very slowly (scan time of approximately 1 h). Surface SIMS is therefore typically used as a surface sensitive analysis technique. Dynamic SIMS (high ion current densities of approximately 1 mA cm^−2^) repeatedly rasters the sample surface, thus creating a depth profile of the analysed film.

Depending on the type of setup used for MS, the measurable mass range can go up to 500 000 amu.^88^ Such a large mass range is extremely advantageous; thus, SIMS is classified as one of the most sensitive analytical techniques. Due to the nature of the material removal process, surface SIMS is considered to be quasi non-destructive (a low volume, between 10 μm^3^ and 1000 μm^3^, is destroyed during analysis) and has been used to perform elemental imaging on low-dimension nanostructures to give insights of the elemental cross-section through depth resolved profiling.^89^

The main disadvantage of MS is the difficulty in the quantification of the analysed data. The reason is that the ionisation probability of the measured species is influenced by multiple variables, such as the composition of the sample, the surface coverage, the kinetic energy, and deciding which species to include in the analysis (*e.g.*, positive, negative or neutral ions). One way to reduce the number of variables is to use calibration standards. However, the standards must possess similar properties to the measured sample, for example similar chemical components, similar dopant concentration and similar ion etch properties. Thus, the most effective method to evaluate the composition is through the qualitative comparison of similar samples.^90–92^

It is worth noting that the removal of atoms and ions from the film surface with a highly energetic (>1 kV) ion beam causes a collision cascade into the further layers, thereby promoting internal mixing and consequently damaging the film. Therefore, the lateral and depth resolutions are affected. Due to this phenomenon, the depth resolution is generally between a single unit cell and a few nanometres. The resolution is further dependent on the mass of the incident ion. Atoms with a high mass (*e.g.*, Cs with *m* = 133) are capable of reliably attaining high depth resolution, while minimising the sputter-induced damage.

As described in Section 2.1, the pressure impacts the mean free path of the species to be analysed. Therefore, in order to reduce collisions during the time of flight to the detector, both the MS and the analyte housing must be kept in an ultra-high vacuum, as lower pressures improve the signal to noise ratio.

The MS is composed of an ion source, a set of lenses to direct the ion beam, a mass separator, and a detector. Depending on the type of experiments, the ions either originate directly from the sample through the formation of a plasma, or an external tool is used to ionise the collected species, either an ion gun or an ionisation cage. The mass separator creates an electric field to discriminate ions based on *m*/*z* before reaching the detector, meaning that doubly ionised species will also be detected at half the mass. Thus, these possible sources of interference must be considered for data analysis.

**Fig. 20:**
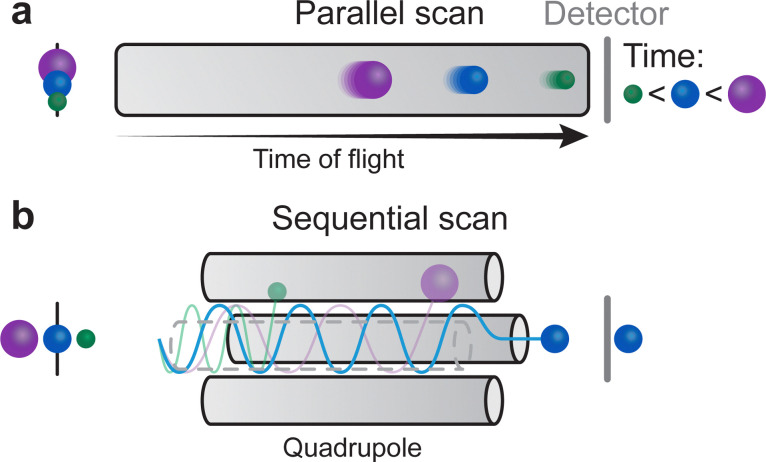
Schematic demonstrating the operating modes of (a) time of flight and (b) quadrupole mass spectrometry.

To analyse the species of a laser induced plasma, the two most common types of spectrometers for PLD applications are quadrupole MS and time-of-flight MS. The time-of-flight MS (Fig. 20(a)) analyses ions based on the time required to travel to a detector under an applied electric field. The time required to reach the detector is therefore mass dependent. Analyses are done in parallel, as ions with differing *m*/*z* can reach the detector concurrently. In quadrupole MS (Fig. 20(b)), a set of electrostatic lenses creates an electric field that directs the ion beam in both the energy and mass analyser before reaching the detector. The ions are sorted based on their energy and their mass, as an ion with an energy or a mass outside of the limits set by the instrument cannot reach the detector. This is a sequential study where only one *m*/*z* ratio is analysed at a time.

MS is tremendously useful in understanding the fundamental aspects of the PLD process. This technique finds uses both in the analysis of the plasma plume and in the analysis of the resulting films. A narrow MS endcap can be utilised to analyse the ablation plume. The kinetic energies of the plasma species can be observed by an MS equipped with both an energy and a mass analyser. Such experiments have been described by Ojeda *et al.*^26^ for mapping the kinetic energies of the plasma with angles of up to 80° with respect to the MS axis. This technique can be used both *in situ* and *ex situ* for the analysis of grown films, depending on the facilities available. One of the limitations of the MS is the difficulty to perform quantitative analysis. Thus, most users will opt for comparative studies of a series of samples. This technique can be used to investigate the interfaces, intermixing, elemental diffusion or depletion, and tracking isotopes. It can also be used for elemental imaging, as well as two-dimensional and three-dimensional depth profiling. As an *in situ* technique, it can be equally useful for the monitoring of film growth through analysis at different stages of the growth. This analysis provides an understanding of how the different PLD parameters discussed in this review can affect the film growth for a particular composition.

Sputtered neutrals MS has been proposed as a method to resolve the limitation of quantification in MS. In this technique, the study of the analyte is independent of the ionisation probability. Thus, the counts observed by the detector reflect the actual composition of the different species within the sample. However, an ionisation cage must be used for sputtered neutrals MS to ionise the neutrals picked up by the endcap of the MS.

### 4.4. Plasma imaging and spectroscopy

Plasma imaging is an *in situ* tool for plasma expansion analysis. The photoemission of excited species from the plasma plume is captured by a set of lenses to understand the plume dynamics. Laser induced fluorescence is another tool similarly employed for learning about the plasma dynamics.^93^ Plasma imaging allows the investigation of excited species already present in the plasma as a product of collisions and reactions, and gives crucial pieces of information regarding the composition and fragmentation of the plasma. In laser induced fluorescence, a single wavelength light source (laser) is used to excite the species contained in a plasma, consequently observing their fluorescence signal. Monitoring the temporal and spatial evolution of plasma species provides insights into the plasma expansion dynamics, which enables an enhanced understanding of the correlations between PLD parameters and the film growth. For additional details on these techniques, as well as comparisons of the limitations, readers are directed to the work of Harilal *et al.*^94^ Here, we will discuss the practical aspects of *in situ* plasma plume analysis.

**Fig. 21:**
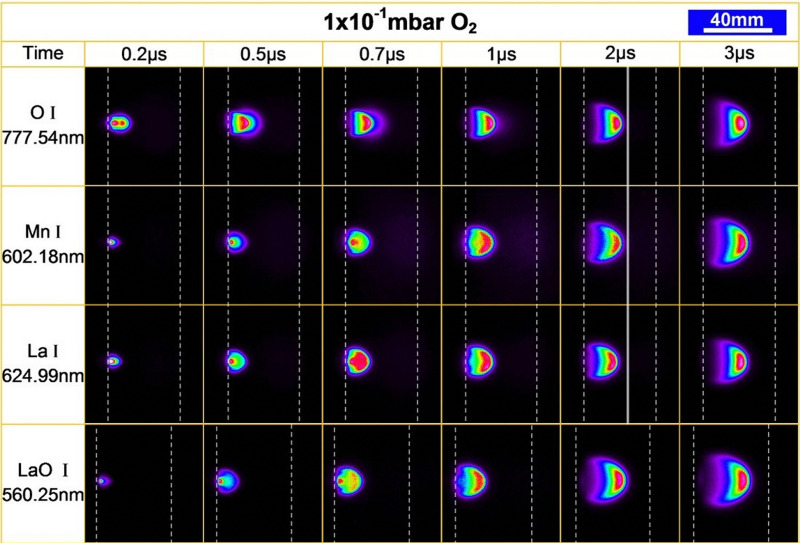
Time resolved images tracing the spatial distribution of the excited species of O I, Mn I, La I and LaO I. The intensity scale has been normalized to the maximum counts for each frame, due to a ten-fold decrease in intensity between 0.2 μs and 3 μs. The use of a differing acousto-optic tunable filter for LaO I results in a slightly altered frame and zoom and therefore cannot be compared directly with the other species. Reproduced from ref. 26 with permission from ETH Zürich, copyright 2016.

For plasma spectroscopy and imaging, the most common method is to first measure a part of an excitation spectrum to investigate which excited species (neutrals or ions) are in the plasma and from which excited state the photon originates. Due to the finite lifetime of the excitation (between approximately 10 ns and 1 μs), the temporal and spatial evolution of these excited states can be monitored. Selected elements can be tracked by utilising specific line filters, thereby obtaining time and space resolved information for individual elements. Likewise, element selective images of the plume species can be observed by projecting the excitation line directly onto a diffraction grid, which additionally acts as a mirror. However, the use of a high-speed camera equipped with an intensified CCD (ICCD), either with line filters or in combination with an acousto-optical tuneable filter, is an approach that provides greater flexibility. This combination enables spatial, temporal, and species-resolved studies of the plume expansion on the time scale between nanoseconds and microseconds. The added benefit of the acousto-optical tuneable filter is the freedom to select the filter wavelength between 350 nm and 800 nm, albeit at the expense of a reduced linewidth. This way, the temporal and spatial propagation of excited species in the plume can be studied, limited only by the wavelength sensitivity of the ICCD in terms of the time resolution. As an example, such an analysis is demonstrated in Fig. 21. Here, the expansion of optically excited neutrals of O, Mn, La and LaO have been tracked for 3 μs during the ablation of a La_0.4_Ca_0.6_MnO_3_ target. The expansion of the excited species up to 0.7 μs is related to the mass and most visible in the O I. However, the differences quickly disappear, and a collective front is consequently observed for all recorded species (shown by the vertical line at 2 μs in Fig. 21). This translates into a simultaneous and very slow (approximately 650 m s^−1^) arrival of all species at the substrate. The LaO I emission shows a larger area of light emission towards the end of capture, which corresponds to an increase in chemical reactions. These measurements highlight the mass differences in expansion properties and provide insight into the chemical reactivity between the plasma species and the reactive background gas.

**Fig. 22:**
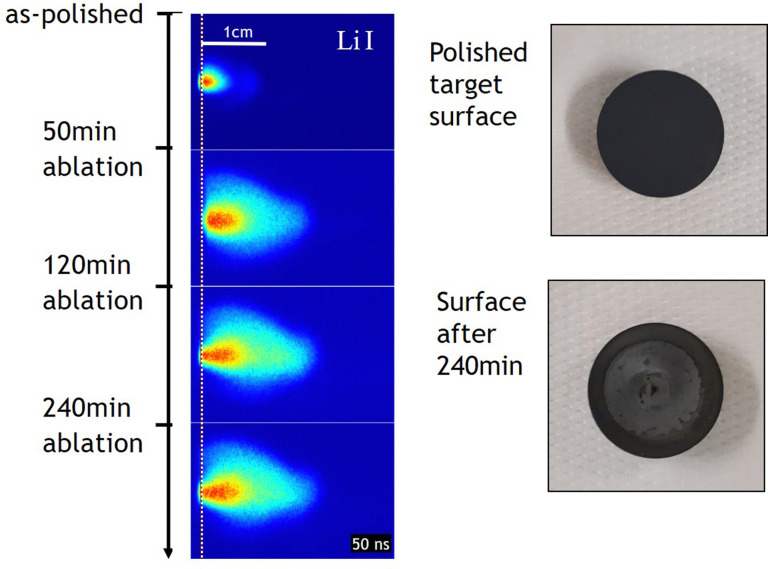
Li I plasma imaging profiles in a LiMn_2_O_4_ target following polishing as a function of ablation time (left). Photographs of the target surface before and after ablation (right). Reproduced from ref. 95 with permission from ETH Zürich, copyright 2022.

On a more practical note, plasma imaging can also be used to monitor the state of a target surface during ablation. As already outlined, the intense laser beam melts the target material and part of the target material can evaporate. For a multi-elemental target, the melting can be congruent as well as incongruent, and the vapour pressure frequently differs between elements, resulting in changes to the surface-near composition of the target as a function of ablation time. This is illustrated in Fig. 22, where the plasma expansion of Li I is monitored (50 ns after laser impact) for a LiMn_2_O_4_ target ablated at a constant fluence for up to 240 min.

The spatial Li expansion (Fig. 22) is significantly higher in the target surface ablated for 50 min, 120 min and 240 min, in comparison to the ground and polished surface, due to changing absorption properties in the target material. Interestingly, the deposition rate of films grown using a LiMn_2_O_4_ target changes slowly with ablation time, which is not observed for targets with stable surface composition. The target surface becomes more compact (dense) following impact by the laser pulses, developing a molten and recrystallised layer on the surface. The repeated ablation of the target increases the roughness of the surface, resulting in an increased surface area, which reduces the energy density at the target. These two effects have opposite consequences on the ablation and deposition rate; however, it is difficult to know which phenomenon dominates throughout the ablation process.

When ablation-induced effects are evident, such as those shown in Fig. 22, one of two possible approaches must be consistently employed for reproducible thin film growth: (1) ablating a freshly polished surface, which requires grinding and polishing the target between each deposition, or (2) reaching a stable surface state prior to deposition, either *via* sufficient pre-ablation or reduced polishing of the target between depositions. The former assumes the surface composition will be reset before each film is prepared and the stoichiometry of the target will be pseudo-constant throughout the necessary ablation timespan. Conversely, the latter assumes that the surface-near composition of a clean target surface is a runaway parameter and therefore requires stable surface stoichiometry and roughness to begin depositing films. Commonly, the choice of approach is guided by the required film thickness, which is dependent on the number of pulses and therefore the ablation time.

## 5. Conclusions and perspective

PLD is a tremendously useful tool for the understanding of material properties at the nanoscale, which in turn enables advances in understanding useful for many emerging technologies. The extent of tuneability of the growth parameters present in PLD presents the possibility for a detailed level of control over the crystallographic, structural, chemical and physical properties of the films, thus impacting their functional characteristics. This control allows for elegant studies, presenting complex relationships and uncovering new phenomena.

The multivariate nature of this technique, which allows for a high degree of tuneability, commonly results in difficulties for the growth of new material classes. Optimisation of the growth parameters is often an iterative approach, performed through the analysis of relevant properties following the film growth, which then forms the basis for further optimisation. This becomes an arduous, time and resource consuming task, especially for those new to the technique. In this tutorial review, we have presented an overview of the technique together with a brief outline of the fundamental concepts necessary to perform first growth experiments. We have provided an extensive discussion on the general parameter-property relationships encountered within PLD. Notably, due to the vast number of compositions possible to grow, it is impossible to provide detailed information on particular compositions, thus general relationships were favoured based on chemical, mass and size trends of the atomic constituents comprising the desired composition. These relationships are aimed as a springboard to begin the optimisation of a new chemical system.

Commonly, the measurement of properties is performed *ex situ*, which can result in significant delays in optimising the growth parameters for a material class. Examples of this are X-ray diffractometry and transmission electron microscopy for structural properties, and Rutherford backscattering spectroscopy and elastic recoil detection analysis for chemical composition. When dealing with epitaxially grown films, the former two techniques can provide information on the epitaxy and the strain behaviour. We have discussed and explained *in situ* monitoring techniques, which provide information on these properties either in real time during the deposition or, in the case of SIMS, can be performed directly following growth. Such information is immensely helpful in the growth optimisation of a new material class.

PLD has become a mature technique in recent times. Previously, the chambers used for thin film growth *via* PLD were often purpose-built by the research teams, which resulted in deviations in the layout and had consequences of reproducibility between chambers. Now, multiple companies are producing commercial PLD chambers (*e.g.*, Neocera, Twente Solid State technologies, PVD Products, PreVac and Surface Systems and Technology, among others). This allows users to exchange growth parameters between similarly-built chambers and requires reduced optimisation to tailor the epitaxial growth of highly crystalline materials. Nonetheless, many challenges still remain in the development of the technique.

One of the key limitations has traditionally been the low throughput capability of the technique, which in turn limits the applicability of PLD in commercial settings. The laser ablation limits the spot size and/or the fluence on the target, thus forming a relatively small plume diameter, limiting the substrate area with a homogeneous thickness distribution to a maximum of a few square centimetres. A solution to this problem requires at least one of the following approaches: (1) a spot size higher than a few square millimetres with a homogeneous laser energy profile, and sufficient fluence to ablate the target and form a plasma plume; (2) a sufficiently high deposition rate, which can possibly be achieved with an increased laser pulse repetition rate; and/or (3) the capability to intelligently scan over a larger substrate surface in such a manner as to produce a homogeneous thickness and composition distribution.

The concept of producing large area coatings is one that dates back to the push for high temperature superconductors. A historical perspective and an extensive discussion on large-area PLD is discussed by Greer.^96^ Two possible directions were identified, either the scanning of the laser beam on the target surface, or the scanning of the substrate relative to the plasma plume. As the homogeneity of the laser intensity profile is crucial, the latter approach has become more widely accepted. For example, Solmates has recently demonstrated a below 0.3% thickness variation over a 300 mm wafer surface utilising this method. One of the largest challenges in this space is the intelligent path control. With the rise in machine learning implementation in production, such feedback control can be employed together with *in situ* quantification methods, depending on the required control type. As an example, thickness determination can be incorporated into the feedback loop, resulting in improved thickness homogeneity through re-deposition in deficient regions.

Increases in spot size for the purpose of coating large area substrates have been achieved by widening the laser beam optically in one direction. This method can be used to ablate a horizontally mounted cylindrical target, such that the long axis of the beam corresponds to the long axis of the target. This approach has achieved large area coatings on substrates with dimensions at 20 cm by 20 cm, showing sufficient homogeneity and stoichiometric chemical composition.^8–10^ This is an ongoing endeavour, with the recent demonstration by Coherent of the combination of multiple lasers using optics, whereby a pulse energy of up to 6 J can be achieved by employing three lasers, with a homogeneous laser intensity area of up to 9 cm^2^ (1500 mm length and 0.6 mm width) and a frequency of up to 600 Hz; however, it is worth noting that these values correspond to a wavelength of 308 nm.

Achieving high frequencies in lasers is another ongoing challenge. High repetition rates are limited by supersaturation and the growth mode of the deposited material. For nanosecond lasers, the effects of ablation frequencies in the kHz range on the growth dynamics of thin films are not yet clear. While currently it is more promising to scan the laser over the target to coat large area substrates,^96–98^ we anticipate the improvements in repetition rate of the laser to further aid in transitioning PLD towards commercial applications.

One possible application of PLD with recent research efforts is combinatorial pulsed laser deposition. This subject has recently been discussed by McGinn^99^ and von Wenckstern *et al.*,^100^ and readers are directed to these for further information. Here, multiple complementary chemical compositions can be combined to form solid solutions with well-defined compositional gradients. This is commonly performed using multiple targets with complementary chemical compositions. Such a technique is capable of rapidly determining composition–property relationships and consequently creating binary, ternary and quaternary phase diagrams for complex cation stoichiometries. The possibility to combine this technique with *in situ* compositional analysis is anticipated to provide an additional layer of understanding of the relationship between composition and properties of materials. Combinatorial PLD is therefore a tremendously useful tool, which we believe will accelerate progress in identifying optimal compositions for particular functional properties.

Another promising application of PLD with rapidly increasing interest is high entropy materials. The combination of multiple cationic species occupying a single site in crystalline materials has the potential to alter its structural, physical and functional properties through chemical disorder. PLD is the optimal technique for the deposition of such materials, taking into account the unique benefit of the technique to transfer highly complex chemical compositions from the target to the substrate. Initial results have demonstrated potential in a multitude of applications. For example, high temperature superconductors have recently been demonstrated with high entropy in the rare earth cation site for REBa_2_Cu_3_O_7−*δ*_ films^101^ and electrocaloric properties have been shown in lead zirconium titanate thin films with disorder in the b-site cation.^102^ This is an exciting development and we anticipate new phenomena will be uncovered by the use of such high entropy materials.

## Conflicts of interest

There are no conflicts to declare.

## Supplementary Material
