# Correction: A practical guide to pulsed laser deposition

**DOI:** 10.1039/d6cs90021f

**Published:** 2026-03-13

**Authors:** Nick A. Shepelin, Zahra P. Tehrani, Natacha Ohannessian, Christof W. Schneider, Daniele Pergolesi, Thomas Lippert

**Affiliations:** a Laboratory for Multiscale Materials Experiments, Paul Scherrer Institut CH-5232 Villigen Switzerland nikita.shepelin@psi.ch thomas.lippert@psi.ch; b Department of Chemistry and Applied Biosciences, ETH Zürich CH-8093 Zürich Switzerland lippertt@ethz.ch

## Abstract

Correction for ‘A practical guide to pulsed laser deposition’ by Nick A. Shepelin *et al.*, *Chem. Soc. Rev.*, 2023, **52**, 2294–2321, https://doi.org/10.1039/D2CS00938B.

The authors regret that there is an error in Fig. 3 in the original article. The values for the mean free path are incorrect by two orders of magnitude, which stemmed from a missing pressure conversion from Pa to mbar (1 mbar = 100 Pa) in panel b only. Consequently, the mean free path (Fig. 3b) should be reduced by two orders of magnitude for a given pressure value. The slope of the line remains the same. An updated version of Fig. 3 is provided here.

**Fig. 3:**
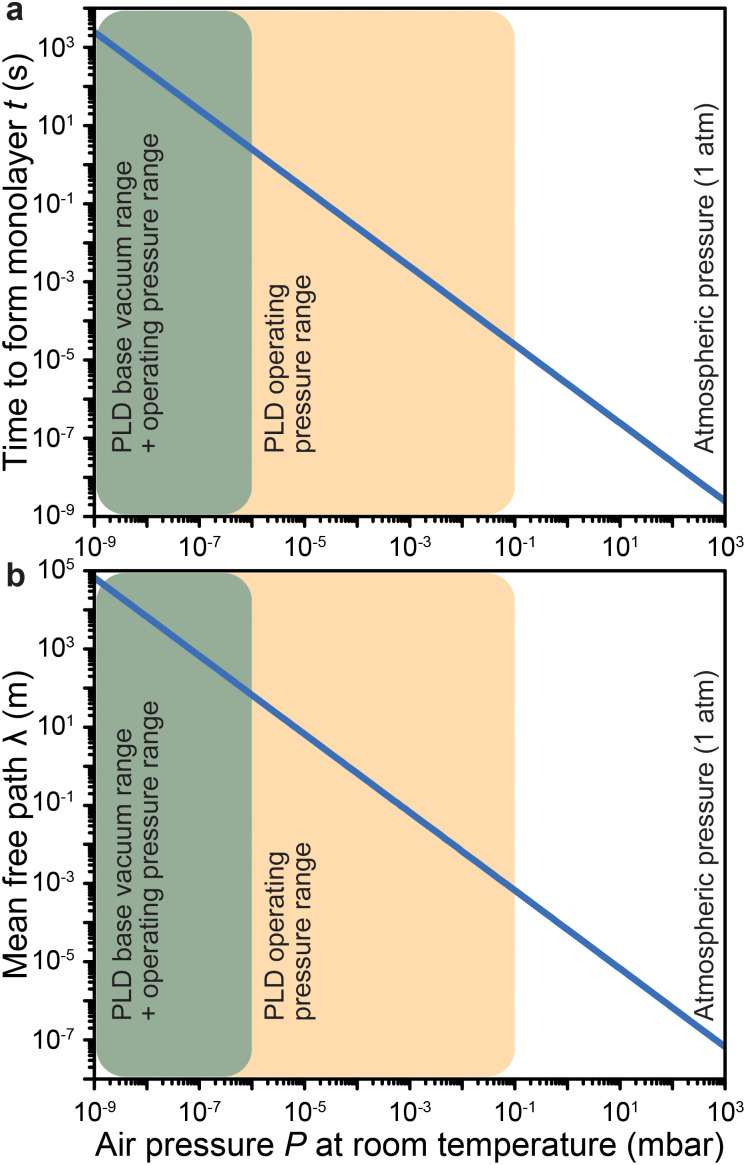
The impact of air pressure at room temperature on (a) the time to form a monolayer of adsorbate species that are present in the vacuum chamber and (b) the mean free path. The yellow overlay corresponds to the pressure range over which deposition of films has been reported. The green overlay corresponds to typical base pressures of pulsed laser deposition. Film growth can also proceed in the latter range.

Eqn (3b) should also be updated as follows:
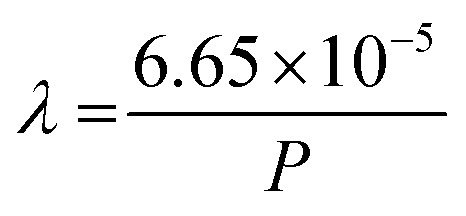


Finally, the text below eqn (3b) should be updated to read: “As shown in Fig. 3(b), the environmental pressure drastically influences the mean free path, ranging in scale from tens of nanometres to tens of metres by decreasing the pressure from atmospheric values down to 10^−6^ mbar.”

The Royal Society of Chemistry apologises for these errors and any consequent inconvenience to authors and readers.

